# Phytochemicals of *Vitis vinifera* L. var. King Ruby protect mice from benzo(a)pyrene-induced lung injury

**DOI:** 10.1038/s41598-025-86173-x

**Published:** 2025-02-06

**Authors:** Gehad S. Ahmedy, Hend M. Selim, Mona El-Aasr, Souzan M. Ibrahim, Suzy A. El-Sherbeni

**Affiliations:** 1 Department of Pharmacognosy, Faculty of Pharmacy, Tanta University, Tanta, 31527 Egypt; 2 Department of Biochemistry, Faculty of Pharmacy, Tanta University, Tanta, 31527 Egypt

**Keywords:** *Vitis vinifera*, Benzo(a)pyrene, Lung protection, LC–ESI–MS/MS, Inflammation, Polyphenols., Cancer, Biochemistry, Plant sciences, Cancer, Lung cancer

## Abstract

The world’s concern about smoking hazards, chronic obstructive pulmonary disease (COPD) and lung cancer was the motivation to investigate plants as a source of new drugs with lung protective effect. The phytochemical profile of *Vitis vinifera* L. var. King Ruby leaves methanol extract (VLME) was tentatively recognized by liquid chromatography electrospray ionization tandem mass spectrometric (LC-ESI-MS/MS). Fifty-two and forty-seven compounds were identified by negative and positive ESI modes, respectively. Taraxerol **(1)**, *β-*sitosterol **(2)**, daucosterol **(3)**, quercetin-3-*O*-*β*-D-glucuronoide-6″-methyl ester **(4)** and isoquercetin **(5)** were isolated from VLME. The sulforhodamine B (SRB) assay of the different fractions against A-549 cell line revealed that the methylene chloride fraction (MCF) had the lowest cell viability at 300 µg/mL (4.54 ± 0.19%). Mice of 10 groups (*n* = 6) was treated as follows: **Group I** (negative control group), **group II** (disease control, mice received B(a)P 125 mg/kg, orally), **groups III-V** (mice received 100, 200, and 300 mg/kg of VLME, followed by B(a)P), **group VI** (mice received only 300 mg/kg of VLME), **groups VII-XI** (mice received 100, 200, and 300 mg/kg of MCF, followed by B(a)P), **group X** (mice received only 300 mg/kg of MCF). On the seventh day, all groups received a single oral dose of B(a)P 125 mg/kg body, except group **I**,** VI and X**. In vivo studies showed VLME and MCF (300 mg/kg body weight) effectively mitigated benzo(a)pyrene-induced lung injuries in mice. The anti-inflammatory effects were confirmed by the downregulation of cyclooxygenase-2 (COX-2) and CD34, alongside reduced nuclear factor-kappa B (NF-κB) expression. Antioxidant activity was indicated by decreased malondialdehyde (MDA) levels and inducible nitric oxide synthase (iNOS) expression with the remarkable increase in glutathione (GSH). Histological improvements further support the potential of *Vitis vinifera* L. leaves as a natural lung protectant. Further pre-clinical and clinical investigations will be required to deliver a new drug with promising protection effect.

**Supplementary Information:**

The online version contains supplementary material available at 10.1038/s41598-025-86173-x.

## Introduction

Lung-related diseases rank among the leading causes of mortality globally. Smoking tobacco is one of the main causes of lung pathology such as chronic obstructive pulmonary disease (COPD) and lung cancer. The World Health Organization (WHO) estimates that tobacco use causes 1 in 10 fatalities globally and about 7 million deaths annually^1^. The main constituent of tobacco is benzo(a)pyrene [B(a)P], which is linked to several lethal aspects that ​contribute to the​ progression of lung cancer^2^. [​B(a)P] is one of polycyclic aromatic hydrocarbons (PAHs), which is derived by incomplete organic waste combustion, including rubbish, plant degradation, and fossil fuels^3^. Its lipophilic nature makes it suitable for the easy absorption by biological membrane causing pulmonary inflammation, edema and surfactant dysfunction in lungs^4^. B(a)P is metabolized by cytochrome P450_1A1_ enzyme to B[a]P-7,8-diol-9,10-epoxides (BPDE) and *O*-quinones by dihydrodiol dehydrogenases, which induce reactive oxygen species (ROS), resulting in oxidative stress induced DNA, lipids and protein damage^5,6^. B[a]P can be absorbed through inhalation, ingestion, and skin contact, ultimately resulting in eye and skin irritation, as well as lung damage and inflammation. Genetic mutations and chromosomal damage by B(a)P have been shown by earlier studies. Short-term exposure-induced detrimental effect of B(a)P on lungs has made it to establish a model for studying adverse effects on pulmonary system^7^.

Recently, scientists have shown their keen interest to combat chemicals-triggered organs toxicity using herbal and dietary agents rich in alkaloids, flavonoids, terpenes and polyphenolic compounds^8^.

Regulation of oxidative stress is one of the key mechanisms of inflammation, through which it can lead to different cellular damage^8,9^. As a result of releasing ROS and hence oxidative stress, transcription factors such as nuclear factor kappa B (NF-κB), are activated which brings out the secretion of proinflammatory cytokines including both tumor necrosis factor-*α* (TNF-*α*) and interleukin-6 (IL-6), which promotes the activation of inflammatory marker cyclooxygenase-2 (COX-2)^10^.

Our aim of study is to investigate the leaves of the grapes, which belong to the Vitaceae family and are known scientifically as *Vitis vinifera* L, including many varieties such as King Ruby seedless, one of the most widely produced grapevine varieties worldwide^11^.

Due to their remarkable regenerative capacity, grapes were once symbolized as representing life and are occasionally referred to as the “tree of life”^12^. Grapes are consumed in traditional medicine in several nations, such as Pakistan, Italy, and Turkey. They are used as a medicinal treatment for a variety of ailments, including bronchitis, allergies, constipation, digestive aid, colds, flu, anemia, wound healing, and allergies^13^.

Grape leaves contain several phytoconstituents, including derivatives of hydroxycinnamic acid, coumarin, dihydrochalcone, and anthocyanins^14^. Furthermore, the primary constituent of grapes, stilbenoids, exhibit great potential for preventing and treating chronic aging-related diseases^15^. There are several health benefits associated with grape leaves, which include antibacterial, anti-inflammatory, hepatoprotective, gastroprotective, antiviral, antioxidant, antifungal, and antidiabetic abilities^16^.

The massive amounts of leaves, generated by the extensive use of grapes, as well as the bioactivity of their phytoconstituents^17^ have piqued our interest in studying the leaves of the Egyptian variety King Ruby. The current study includes the in vitro anticancer effect against the lung cancer cell line (A-549) to investigate the most active fraction as well as the total methanol extract (VLME) for in vivo study of the potential protective effect against lung injuries in mice caused by benzo(a)pyrene [B(a)P]. Additionally, the recognition of phytochemical profiling employing LC-ESI-MS/MS and isolation of pure compounds from leaves extract were achieved to explore the underlying mechanisms of action.

## Results

### LC–ESI–MS/MS of VLME

The chemical content of VLME was tentatively identified using liquid chromatography combined with tandem mass spectrometry (LC-MS/MS) technique by positive and negative ESI modes. **Supplementary Table SL1** demonstrates 52 known compounds (negative mode ESI), while **Supplementary Table SL2** demonstrates 47 known compounds (positive mode ESI) of different chemical ontologies, including flavones, flavonols, flavanones, catechins, hydroxycinnamic acids, coumaric acids, their glycosides and alkaloids. Figures SL1-SL2 in Supplementary Materials are the TIC of both modes.

### Characterization of flavonoids and their glycosides

The majority of the chemicals found in VLME were flavonoids. Naringenin and apigenin were identified in negative mode with [M − H]^−^ at 271.0587, 269.0443, respectively; also, rhamnetin, kaempferide, and 3′,4′,5,7-tetrahydroxyflavanone were identified in positive mode with [M + H]^+^ at 317.1885, 301.1424, and 289.0718, respectively. Myricetin, taxifolin, luteolin, quercetin, and isorhamnetin were identified in both modes.

The major recognized aglycon in negative mode is quercetin and in positive mode is rhamnetin. The presence of 4′-*O*-methylated flavonoids was also identified in two compounds with [M − H]^−^ at 301.0945 for hesperetin in negative mode and [M + H]^+^ at 285.1477 in positive mode for acacetin.

Vitexin was identified in positive mode as the only *C*-glycoside with [M + H]^+^ at 433.1486 and a peak at 415.16748 for [C_21_H_19_O_9_]^+^^18^, while as *O*-glycosides, 14 compounds were detected in negative mode. Namely, quercetin-3-*O*-arabinoside, quercetin-3-D-xyloside, quercetin-3,4′-*O*-di-*β*-glucopyranoside, marein, hyperoside, narcissin, kaempferol-3-*O*-(6″-p-coumaroyl)-glucoside, astragalin, isoquercitrin, rhoifolin, isorhamnetin-3-*O*-glucoside, phlorizin, kaempferol-3-*O*-*α*-L-rhamnoside, 4-deoxyphloridzin with [M − H]^−^ at 433.0433, 433.0965, 625.1432, 449.1088, 463.0884, 623.1636, 593.117, 447.0944, 463.2183, 577.1517, 477.1019, 435.1287, 431.0999, and 419.2253, respectively.

Nine *O*-glycosides were detected in positive mode. They are isorhamnetin-3-*O*-rutinoside, gossypin, diosmin, hyperoside, quercetin-3-*O*-arabinoside, poncirin, astragalin, naringenin-7-*O*-glucoside, marein with [M + H]^+^ at 625.1744, 481.1043, 609.1943, 465.0999, 435.1619, 595.1608, 449.1102, 435.1341, and 451.1719, respectively. The major recognized glycoside is hyperoside, followed by astragalin in both ion modes.

The most abundant compound in negative mode is miquelianin with [M − H]^−^ at 477.0655, which is also found in positive mode [M + H]^+^ at 479.0815. Also, quercetin-3-*O*-glucuronide 6″-*O*-methyl ester, with [M + H]^+^ at 493.3104. It was isolated for the first time from the ethyl acetate fraction of *Vitis vinifera* L. leaves as compound (4). Kaempferol-3-*O*-glucuronide was also detected in both ion modes, with [M − H]^−^ at 461.0692 and [M + H]^+^ at 463.0854.

### Characterization of isoflavonoid, biflavonoids, stilbenes and catechin

Daidzein-8-*C*-glucoside (puerarin) is *C*-glycoside isoflavonoid that is detected in both ion modes, while ononin is *O*-glycoside isoflavonoid that is found in positive ESI only.

Procyanidin B2 is the only detected bioflavonoids with [M + H]^+^ at 579.1841 in positive ESI mode.

3,3′, 4′,5,7-pentahydroxyflavan is the only catechin detected in both ion modes. Resveratrol is the only stilbene detected with [M + H]^+^ at 229.1538 and [M − H]^−^ at 227.0709, while E-astringin is a stilbene glycoside with [M − H]^−^ at 405.1684 in negative ESI mode.

### Characterization of anthocyanidin and anthocyanidin glycosides

Delphinidin was the only recognized anthocyanidin in negative mode, with [M − H]^−^ at 302.1664 and specific peaks at 257.009 [M − H_2_O–CO]^+^, 229.056 [M − H_2_O–2CO]^+^, 173.031 [M − H_2_O–4CO]^+^^19^.

Tulipanin is an anthocyanidin glycoside that was detected in both ion modes, while cyanidin-3-*O* -glucoside was detected in the positive mode at 449.1079 with a peak at 287.055, thus confirming its identity.

### Characterization of coumarins derivatives and glycosides

Esculin and daphnetin are the coumarins found in the negative mode, with [M + H]^+^ at 339.0726 and 177.0176, respectively. Esculetin and daphnetin were detected in positive mode at 179.0322 and 179.144, respectively. The major one is daphnetin, with fragments of 77.038 [C_6_H_4_] + H^+^, 123.116 [C_6_H_4_O_3_-H]^+^, 133.104 [C_8_H_6_O_2_-H]^+^, 135.116 [C_8_H_6_O_2_] + H^+^^20^.

### Characterization of alkaloid derivatives

Four alkaloids were detected in positive mode: trigonelline, caffeine, nicotinamide, nicotine at 138.0526, 195.0878, 123.0799, 163.1103, respectively. The major one is nicotinamide with fragments at 78.033 [C_5_H_4_N]^+^, 80.049 [C_5_H_4_N + H] + H^+^^18^.

### Characterization of organic acids

In negative mode 3,4-dihydroxyphenylacetic acid, D-(+)-malic acid, maleic acid, D-(-)-quinic acid, (**±**)-jasmonic acid, citraconic acid, chlorogenic acid, *p*-hydroxybenzoic acid, rosmarinic acid, 2,5-dihydroxybenzoic acid and *γ*-linolenic acid were detected with [M − H]^−^ at 167.0345, 133.0137, 115.0032, 191.0205, 209.0665, 129.0182, 353.0879, 137.0244, 359.0789, 153.0176 and 277.2179, respectively. Methyl jasmonate, linoleic acid and chlorogenic acid were also detected in positive mode with [M + H]^+^ at 225.11, 281.1365, 355.1524.

### Characterization of cinnamic acid derivatives

Eight cinnamic acid derivatives were identified in both modes: caffeic acid, trans-ortho-coumaric acid (2-hydroxy cinnamic acid), 3-(4-hydroxyphenyl) prop-2-enoic acid, 1-*O-β*-D-glucopyranosyl sinapate, 3-(4-hydroxy-3,5-dimethoxyphenyl)-2-propenoic acid, and 3-(4-hydroxy-3-methoxyphenyl) prop-2-enoic acid in the negative mode, while in positive mode, 1-*O-β*-D-glucopyranosyl sinapate and trans-cinnamate were identified in the positive mode.

#### Structure identification of the isolated compounds from VLME

Five compounds were isolated from VLME. Compounds **(1)** and **(2)** from pet. ether fraction, compound **(3)** from methylene chloride fraction and compounds **(4)** and **(5)** from ethyl acetate fraction. Their isolation and purification were interpreted in Supplementary Materials with flow charts Scheme S1-S5.

**Fig. 1:**
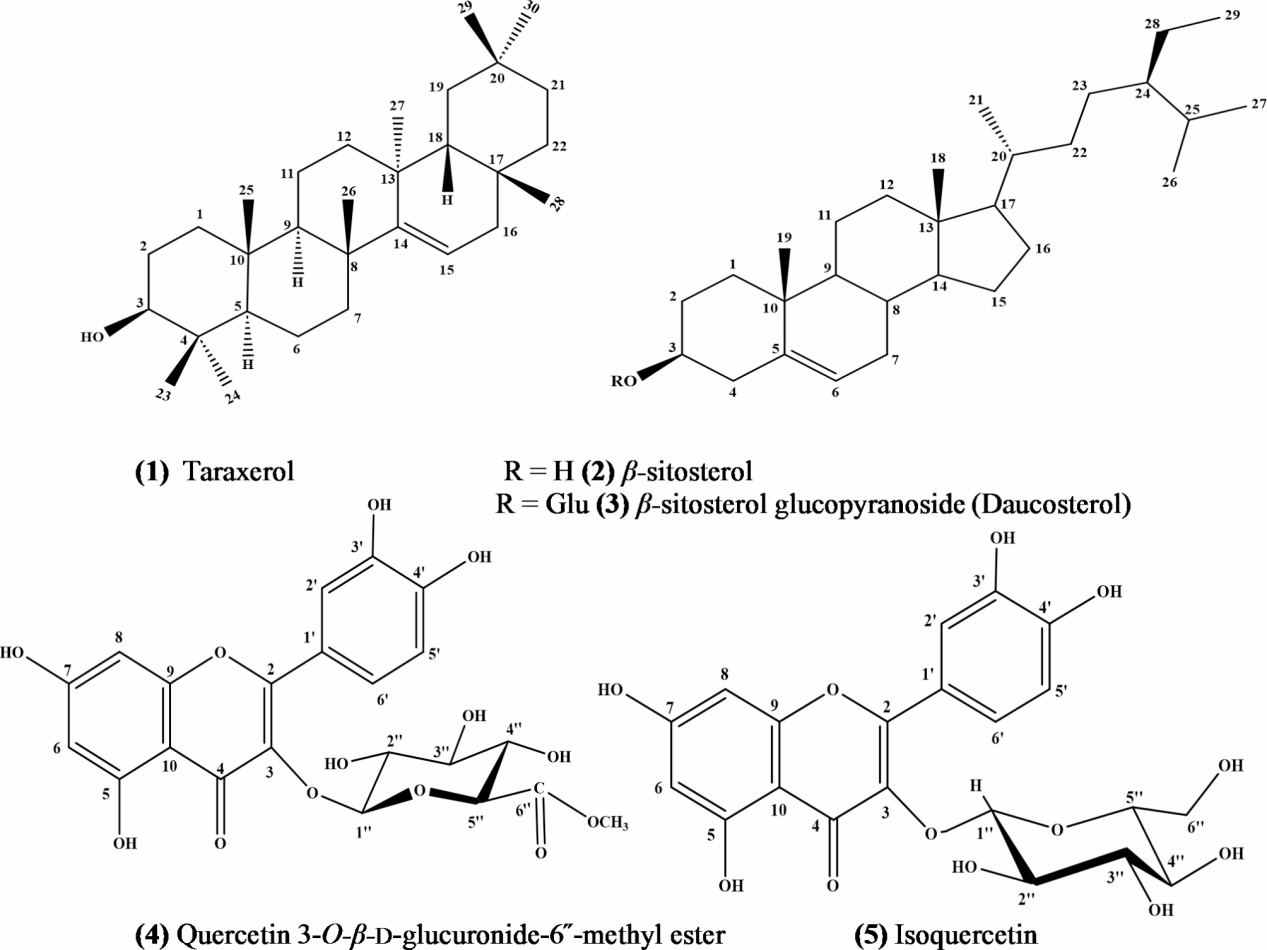
Structures of compounds isolated from leaves of *Vitis vinifera* L. var King Ruby.

Depending on their spectral data, compounds 1–5 were identified as: taraxerol **(1)**, *β-*sitosterol **(2)**, daucosterol **(3)**, quercetin-3-*O-β*-D-glucuronoide − 6″-methyl ester **(4)**, and isoquercetin **(5)**. Their structures are shown in Fig. 1.

According to our knowledge, taraxerol **(1)** and quercetin-3-*O-β-*glucuronoide − 6″-methyl ester **(4)** had first isolated from *Vitis vinifera* L.

### Spectral analysis

Compound **(1)** is a white powder (50 mg), which has an EI-MS *m/z* of 426.38 [M]^+^ with IR, ^1^H-NMR, and ^1^^3^C-NMR data in the Supplementary Materials and Figures S1A-S1D. It was identified as taraxerol after reviewing the literature^21^.

Compound **(2)** is white needles (30 mg), which has an EI-MS of 414.58 [M]^+^ with IR, ^1^H-NMR, and ^13^C-NMR data in the Supplementary Materials with Figures S2A-S2D. It was identified as *β-*sitosterol after reviewing the literature^22^.

Compound **(3)** is a white powder (35 mg), which has an ESI-MS *m/z* of 575.4 [M − H]^−^with IR, ^1^H-NMR, and DEPTQ data in the Supplementary Materials with Figures S3A-S3D. It was identified as daucosterol after reviewing the literature^22,23^.

Compound **(4)** is a yellow powder (55 mg), which has an ESI-MS *m/z* of 491.3 [M − H]^−^ with UV, IR, ^1^H-NMR, DEPTQ-NMR, and HMBC data in the Supplementary Materials with Figures S4A-S4F. It was identified as quercetin 3-*O*-*β*-D-glucuronide-6″-methyl ester after reviewing the literature^24,25^.

Compound **(5)** is a yellow powder (mg), which has an ESI-MS *m/z* of 463.3 [M − H]^−^ with UV, IR, ^1^H-NMR, APT-NMR, and HMBC data in the Supplementary Materials with Figures S5A-S5F. It was identified as isoquercetin after reviewing the literature^24^.

#### In vitro anticancer impact of VLME

**Fig. 2:**
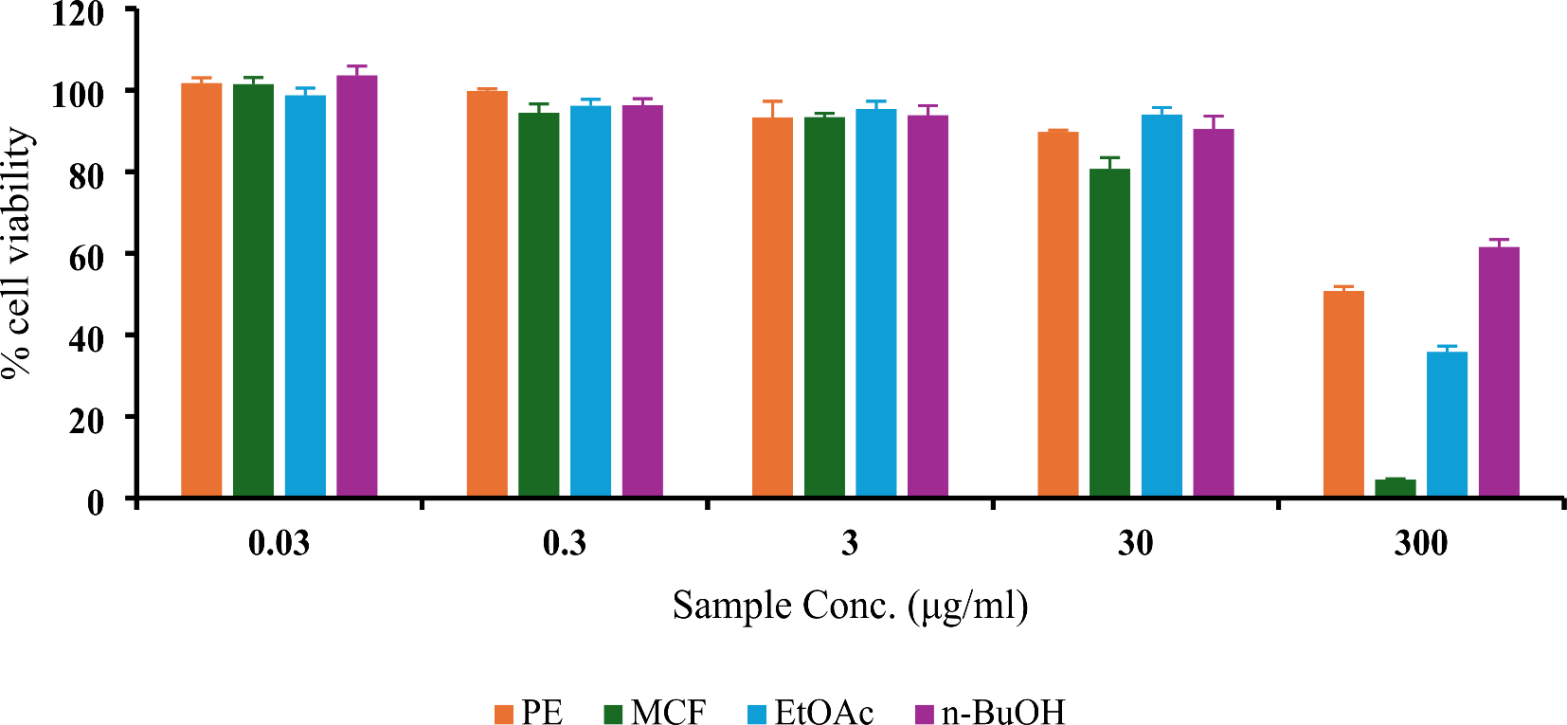
Result of SRB routine multidose cytotoxic assay of the four fractions of VLME on A-549 lung cancer cell line.

- VLME doses (0.03, 0.3, 3, 30, 300 µg/mL) of each fraction (PE, MCF, EtOAc, *n-*BuOH**)** were assessed by sulforhodamine B (SRB) method for anticancer effect against A-549 cell line.
- The data in Table 1; Fig. 2 indicated that all King Ruby fractions showed cytotoxic activity, which is directly proportional to concentration.
- According to % cell viability MCF showed the best activity. Results were expressed as mean ± SD and *p* < 0.05.

**Table 1:** Result of SRB routine multidose cytotoxic assay of the fractions of VLME on A-549 lung cancer cell line.

Sample Conc. (µg/ml)	% Cell Viability			
	PE	MCF	EtOAc	*n*-BuOH
**0.03**	101.76 ± 1.28	101.53 ± 1.58	98.77 ± 1.76	103.651 ± 2.25
**0.3**	99.86 ± 0.45	94.51 ± 2.11	96.20 ± 1.55	96.349 ± 1.59
**3**	93.33 ± 3.93	93.41 ± 0.91	95.40 ± 1.88	93.8798 ± 2.35
**30**	89.78 ± 0.44	80.70 ± 2.79	94.07 ± 1.67	90.4628 ± 3.16
**300**	50.74 ± 1.15	4.54 ± 0.19	35.83 ± 1.43	61.5763 ± 1.81

#### In vivo lung protective activity of VLME

The MCF, which exerted the best effect, was selected for further in vivo lung protective effect against benzo(a)pyrene [B(a)P] induced lung injuries in mice besides VLME.

### Effect of treatments on MDA and GSH levels

The results of **Supplementary Materials**,** Figure ****S1**** (A)** showed that [B(a)P] dosing strongly increased MDA level by 3.2-fold. On the other hand, (VLME) and (MCF) pretreated groups showed a significant suppression in MDA levels (33.13%, 48.9% and 59.5% decrease and 16.4%, 32.9% and 51.8% decrease for 100, 200 and 300 mg of VLME and MCF dosing, respectively), relative to positive control group.

Regarding GSH concentration, **Supplementary Materials**,** Figure ****S1**** (B)** revealed that receiving [B(a)P] caused GSH levels to decrease, significantly (*p* < 0.05, 47.7% decrease), in comparison with normal control. However, pretreating mice with VLME partially corrected GSH levels, when compared to positive control group (1.27, 1.5 and 1.7 fold increase for 100, 200 and 300 mg VLME doses, respectively). Similarly, MCF showed a remarkable increase in GSH level (1.13, 1.23 and 1.5 fold increase for 100, 200 and 300 mg dosing of MCF, respectively), relative to positive control values.

### Effect of treatments on Caspase 3 and NF-ҡB gene expressions

As shown in **Supplementary Materials**,** Figure ****S1**** (A)**: mice received [B(a)P] showed a remarkable increase in apoptotic marker caspase 3 (1.87-fold increase) compared with normal control. On the other hand, groups received 100, 200 and 300 mg/kg of VLME and MCF showed a significant decreased (*p* < 0.05) in caspase 3 gene expression (12.3%, 18.7% and 27.27% decrease and 7.4%, 13.3% and 22.5% decrease, respectively), relative to the positive control.

Likely, pretreating mice with 100, 200 and 300 mg/kg of VLME significantly suppressed NF-ҡB gene expression **Supplementary Materials**, ** Figure ****S2**** (B)** when compared to positive control group (16.06%, 19.17% and 32.6% decrease, respectively). Mice received pretreatment with MCF (100, 200 and 300 mg/kg) displayed a remarkable decrease in NF-ҡB expression as well by 9.3%, 18.6% and 25.4% decrease, respectively) in comparison with the positive control group.

### Histopathological findings

**Fig. 3:**
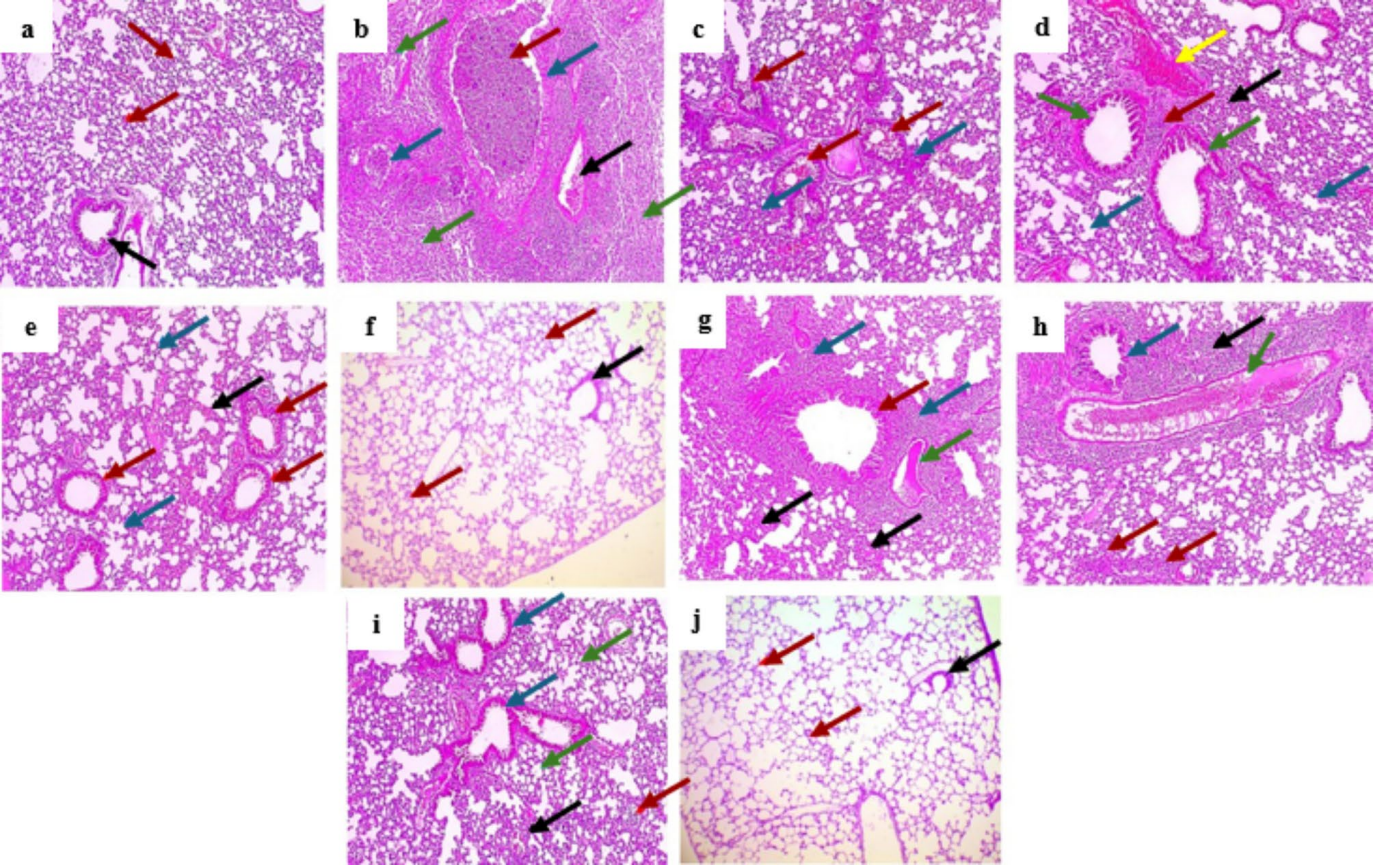
Hematoxylin and eosin (H&E [X 100]) stained lung sections of **(a)** Normal control group (Group I) control group showed normal sized alveoli separated by fibrous septa [red arrows] and normal sized bronchiole (black arrow). **(b)** Group II (positive control, B(a)P) showed dilated destructed bronchioles (blue arrows) with squamous metaplasia and filled with suppuration (red arrow) surrounded by congested blood vessel (black arrow) interstitial inflammation and alveolar fibrosis (green arrows). **(c)** Group III (100 mg VLME **+** B(a)P) showed destructed bronchioles (red arrows) surrounded by destructed alveoli with moderate interstitial hemorrhage, inflammation and fibrosis (blue arrows). **(d)** Group IV (200 mg VLME **+** B(a)P) showed average sized bronchioles (green arrows) surrounded by mild interstitial inflammation (red arrow), congested vessel (yellow arrow), mild focal alveolar fibrosis (black arrow) and most of the alveoli normal sized (blue arrows). **(e)** Group V (300 mg VLME **+** B(a)P) showed average sized bronchioles (red arrows) surrounded by normal sized alveoli (blue arrows) and focal interstitial haemorrhage (black arrow), with no inflammation or fibrosis. **(f)** Group VI (300 mg VLME only) showed terminal bronchiole (black arrow) and fibrous septa [red arrows] separate normal-sized alveoli. **(g)** Group VII (100 mg MCF **+** B(a)P) showed dilated destructed bronchiole with squamous metaplasia without suppuration (red arrow) surrounded by congested blood vessel (green arrow), severe interstitial inflammation (blue arrows) and moderate alveolar fibrosis (black arrows). **(h)** Group VIII (200 mg MCF **+** B(a)P) showed average sized bronchiole (blue arrow) surrounded by moderate interstitial inflammation (black arrow), mild alveolar fibrosis (red arrows) and congested blood vessel (green arrow). **(i)** Group IX (300 mg MCF **+** B(a)P) showed average sized bronchioles (blue arrows) surrounded by mild interstitial inflammation (red arrow), some alveoli showed mild fibrosis (black arrows) and others of normal sized (green arrows). **(j)** Group X (MCF 300 mg only) showed terminal bronchiole (black arrow) and fibrous septa [red arrows] separate normal-sized alveoli.

Compared to sections from the normal control group, the positive control exhibited dilated destructed bronchioles surrounded by congested blood vessels with interstitial inflammation and fibrosis. On the other hands pretreating mice with either methanol or methylene chloride extract showed improved histological findings starting from moderate to mild and ending with weak inflammation and fibrosis on a dose dependent manner **(**Fig. 3**)**.

### Immunohistochemical findings

**Fig. 4:**
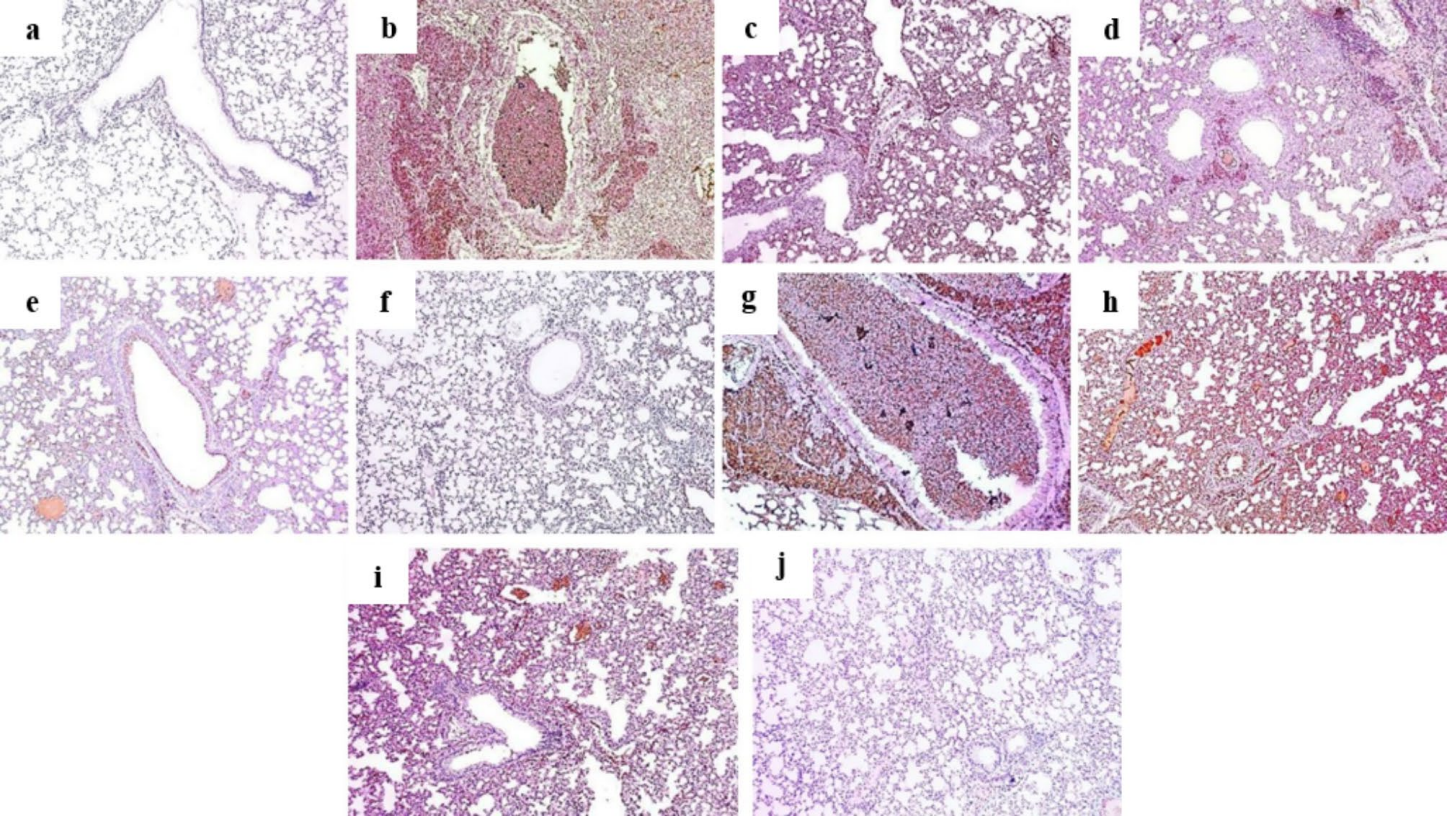
Sections in lung tissues showing COX-2 immunostaining [ X 100] of: **(a)** Normal control group showed negative COX-2 expression (score 0). **(b)** Group II (positive control group, B(a)P) showed marked COX-2 expression (score 4). **(c)** Group III (100 mg VLME **+** B(a)P) showed strong COX-2 expression (score 3). **(d)** Group IV (200 mg VLME **+** B(a)P) showed moderate COX-2 expression (score 2). **(e)** Group V (300 mg VLME **+** B(a)P) showed weak COX-2 expression (score 1). **(f)** Group VI (300 mg VLME only) showed negative COX-2 expression (score 0). **(g)** Group VII (100 mg MCF **+** B(a)P) showed marked COX-2 expression (score 4). **(h)** Group VIII (200 mg MCF **+** B(a)P) showed strong COX-2 expression (score 3). **(i)** Group IX (300 mg MCF **+** B(a)P) showed moderate COX-2 expression (score 2). **(j)** Group X (300 mg MCF only) showed negative COX-2 expression (score 0).

**Fig. 5:**
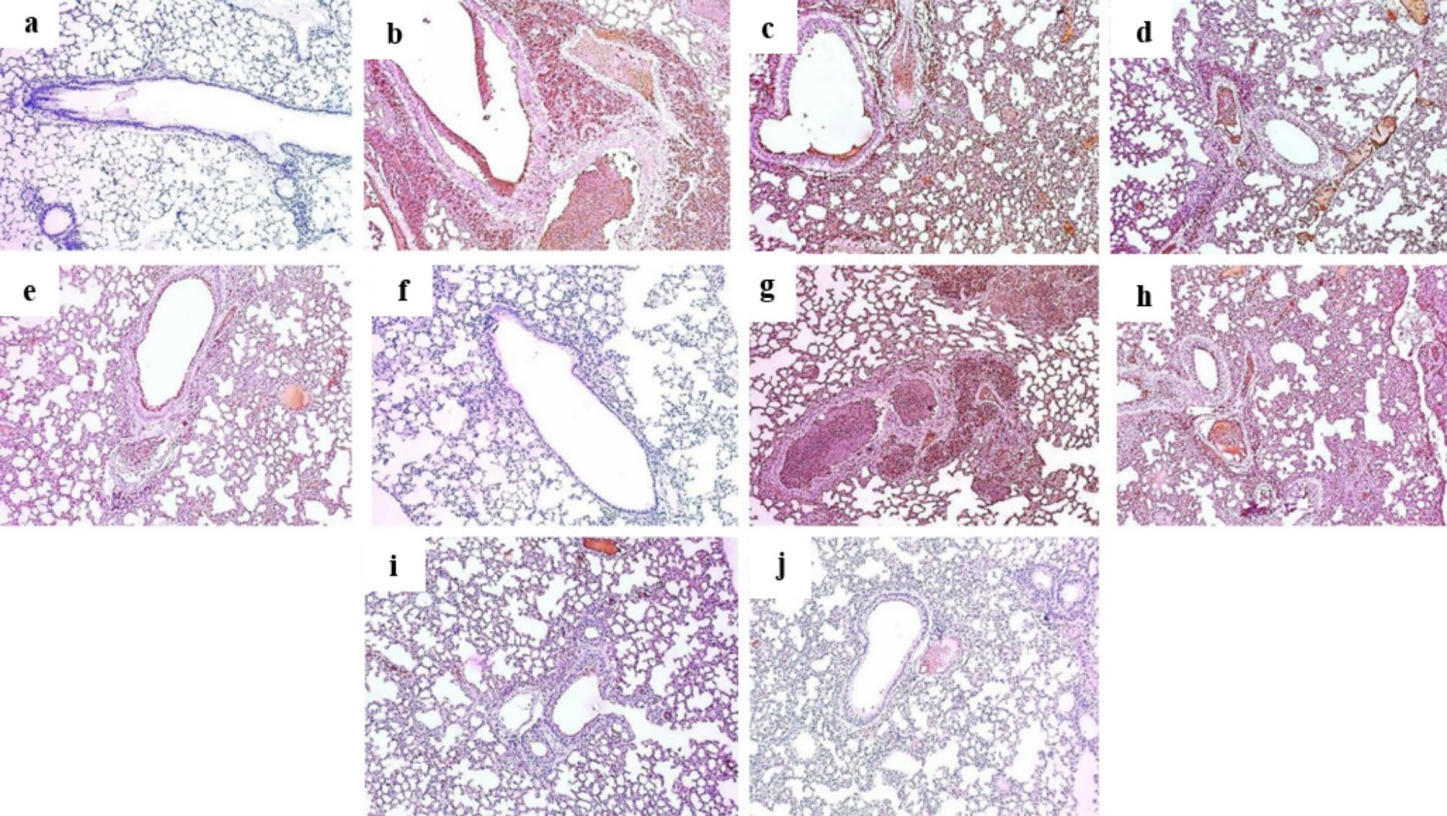
Sections in lung tissues showing CD34 immunostaining [ X 100] of **(a)** Normal control group displayed negative CD34 expression (score 0). **(b)** Positive control group displayed marked CD34 expression (score 4). **(c)** Group III (100 mg VLME **+** B(a)P) displayed strong CD34 expression (score 3). **(d)** Group IV (200 mg VLME **+** B(a)P) displayed moderate CD34 expression (score 2). **(e)** Group V (300 mg VLME **+** B(a)P) displayed weak CD34 expression (score 1). **(f)** Group VI (300 mg VLME only) displayed negative CD34 expression (score 0). **(g)** Group VII (100 mg MCF **+** B(a)P) displayed marked CD 34 expression (score 4). **(h)** Group VIII (200 mg MCF **+** B(a)P) displayed strong CD34 expression (score 3). **(i)** Group IX (300 mg MCF **+** B(a)P) displayed moderate CD34 expression (score 2). **(j)** Group X (300 mg MCF only) displayed negative CD34 expression (score 0).

**Fig. 6:**
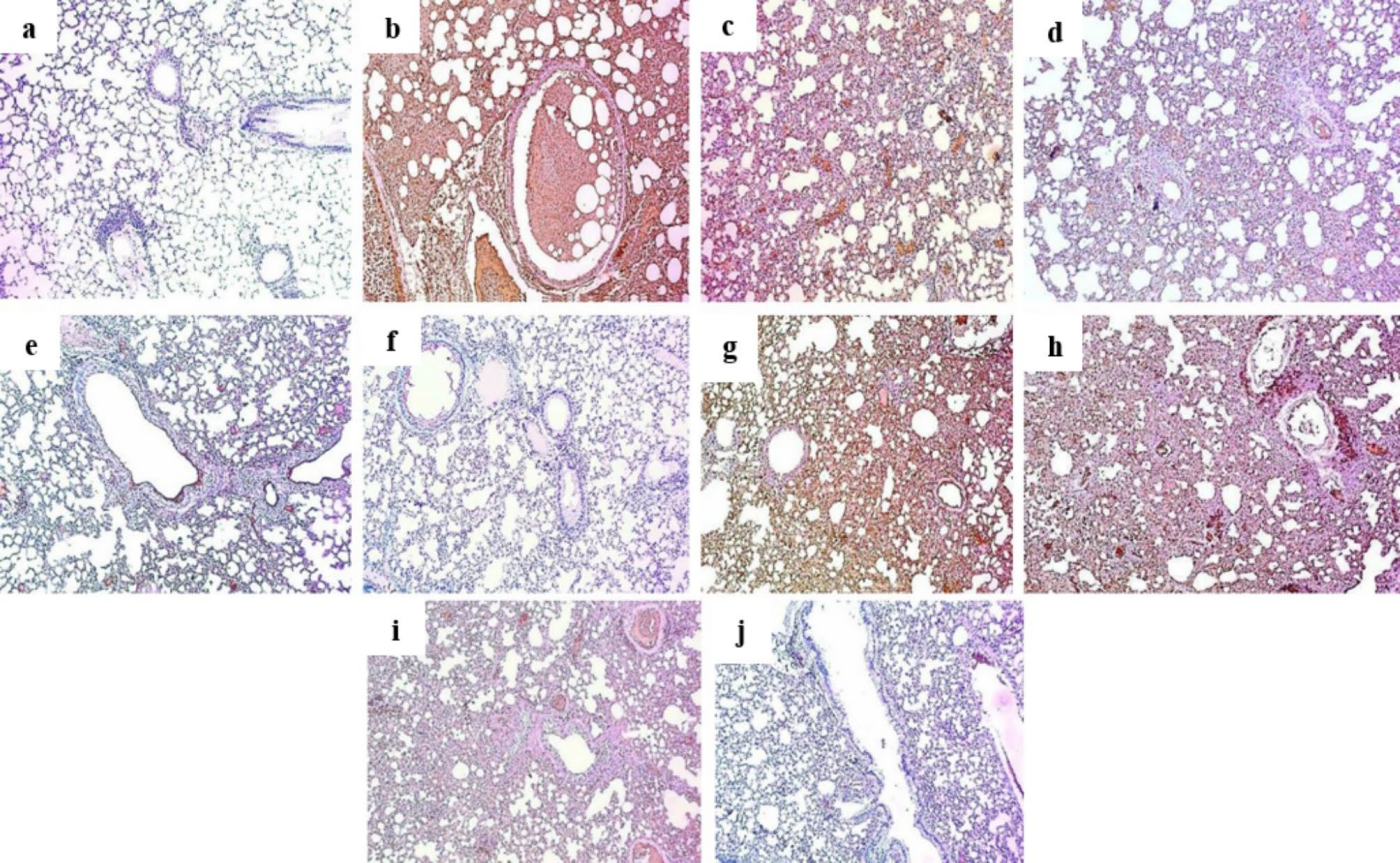
Sections in lung tissues showing iNOS immunostaining [ X 100] of **(a)** Normal control group showed negative iNOS expression (score 0). **(b)** Positive control group revealed marked iNOS expression (score 4). **(c)** Group III (100 mg VLME **+** B(a)P) showed strong iNOS expression (score 3). **(d)** Group IV (200 mg VLME **+** B(a)P) showed moderate iNOS expression (score 2). **(e)** Group V (300 mg VLME **+** B(a)P) showed weak iNOS expression (score 1). **(f)** Group VI (300 mg VLME only) revealed negative iNOS expression (score 0). **(g)** Group VII (100 mg MCF **+** B(a)P) showed marked iNOS expression (score 4). **(h)** Group VIII (200 mg MCF **+** B(a)P) showed strong iNOS expression (score 3). **(i)** Group IX (300 mg MCF **+** B(a)P) showed moderate iNOS expression (score 2). **(j)** Group X (300 mg MCF only) showed negative iNOS expression (score 0).

Pretreating mice before inducing lung toxicity showed an improvement of COX-2 **(**Fig. 4**)**, CD34 **(**Fig. 5**)** and iNOS protein expression **(**Fig. 6**)**, when compared to positive control section. Both extracts revealed a marked decrease in the protein expression scores on a dose dependent manner.

## Discussion

New studies have shown the antioxidant and superoxide-scavenging effects of specific active components in herbal medicine, as well as their inhibitory effects on lipid peroxidation. Some herbal medicines have anti-inflammatory, antipyretic, analgesic, and anti-cancer qualities; they are employed in nutritional supplements^26^. Eating grape products may reduce the probability of breast and colon cancer. This anticancer effect is mostly due to their antioxidant, anti-proliferative, and anti-inflammatory properties^27^.

The LC-ESI-MS/MS investigations of VLME tentatively identified 52 compounds in negative mode and 47 compounds in positive mode. There are different chemical ontologies. Trigonelline, caffeine, nicotinamide and nicotine are alkaloids that were identified by positive mode only. Additionally, tropolone as hinokitiol, sesquiterpene as farnesol, aurone *O*-glycosides as maritimetin 6-*O*-glucoside, and procyanidin B2 were identified in positive mode; these chemical ontologies were not found in negative mode, which confirms that both modes are complementary for identifying the majority of compounds present in the extract.

Major metabolites recognized in negative mode are quercetin-3-*O*-glucuronide, *γ*-linolenic acid, hyperoside and kaempferol-3-*O*-glucoside. Major metabolite recognized in positive mode: 3,3′,4′,5-tetrahydroxy-7-methoxyflavone, choline, 1-*O-β*-D-glucopyranosyl sinapate and hyperoside. Their structures illustrated in Supplementary Materials, Figure SL3.

These compounds possess many activities as anti-inflammatory, anticancer, and antioxidant activities. Quercetin-3-*O*-glucuronide exhibits the ability to protect the lungs by reducing inflammation, and apoptotic cell death. This adds to its ability to prevent the development of pulmonary injuries^28^. Hyperoside has variety of biological actions, including antibacterial, anticancer, antidepressive, antiviral, anti-inflammatory, and organ-protecting abilities Consequently, it can be employed in the treatment of various ailments, including sepsis, arthritis, pulmonary fibrosis, and cancer^29^. Also, several studies have proven the ability of quercetin to increase the effectiveness of anticancer medicines. The anticancer effect of quercetin included apoptosis besides the suppression of cell growth in breast, lung, oral, and prostate cancer^30^. Moreover, quercetin is a possible chemosensitizer since it is an MDR modulator^31^.

Taraxerol **(1)**, *β*-sitosterol **(2)**, daucosterol **(3)**, quercetin-3-*O*-*β*-D-glucuronoide-6″-methyl ester **(4)**, and isoquercetin **(5)** were isolated form VLME. Their structures were determined by reviewing their spectral data with those in the existing literature^21–25^, which discussed in Supplementary Material. To our knowledge, taraxerol **(1)** and quercetin-3-*O*-*β*-D-glucuronoide-6″-methyl ester **(4)** were isolated from *Vitis vinifera* L. for the first time.

Therefore, the in vitro anti-lung cancer efficacy of the four *Vitis vinifera* L. fractions was assessed using the SRB cell viability test on the A-549 lung cancer cell line. The SRB results indicated that the methylene chloride fraction (MCF) exhibited the best activity with % cell viability of 4.54 ± 0.19 at 300 µg/mL. This suggested a potential lung protective effect of MCF of *Vitis vinifera* L against B(a)P induced lung injuries which may lead to lung cancer. Since benzo(a)pyrene, a major carcinogenic component of cigarette smoke, has been linked to the lung cancer associated with the exposure to the environmental tobacco smoke^2^. The Sulforhodamine B (SRB) assay is a colorimetric method used to assess cell density. While it is widely utilized for evaluating cytotoxicity and cell proliferation, particularly in cancer research, there are inherent limitations. So further in vivo studies are needed to figure out the potential anti-lung cancer effect of the leaves of *Vitis vinifera*^32,33^.

The in vivo study examined the possible lung protective effect of VLME and MCF by histologically evaluating the lung tissues and detecting inflammatory and apoptotic markers after short-term exposure of the mice’s lungs to B(a)P, which was used as a model to cause lung injury.

Reactive oxygen species plays a vital role in mediating various pathologies including arthritis and diabetes which are usually accompanied with inflammation. B(a)P is documented to cause genotoxicity through binding to DNA bases and forming DNA adducts as well as breaking DNA strands^4^. The metabolism of B(a)P initiates the formation and release of different ROS like O_2_, −OH, and H_2_O_2_^2^. One of ROS destructive targets is lipid peroxidation, which results in MDA production, which, itself, activates more ROS release and further oxidative stress burst^34^. Herein, mice treated with [B(a)P] revealed a marked increase in MDA levels, while pretreatment with different doses of VLME and MCF revealed a significant decrease in MDA lung levels, proving the possible protective effect of *Vitis vinifera* L. leaves extracts against lung toxicity, in a dose dependent manner, with VLME showing the upper hand over MFC. Our findings were supported by earlier study conducted by^35^, where *Vitis vinifera* L. leaves extract showed antioxidant effect by suppressing MDA levels in carbon tetrachloride-induced liver damage.

Glutathione is a tripeptide antioxidant molecule which acts mainly through two ways, either directly by interaction of –SH group with ROS or indirectly by incriminating ROS detoxification enzymatic reactions. In normal condition, GSH is well balanced and can easily scavenge any ROS production, but in lung toxicity GSH stores are shown to be defected and the level of GSH is strongly decreased^36^. This was confirmed with our results, where [B(a)P] lessened the lung GSH values, whereas mice groups pretreated with both extracts showed improvement of GSH levels, with VLME being powerful than MCF. In line with the present results was a previous study, in which *Vitis vinifera* L. leaves extract speculated to rise GSH levels in rats with testicular damage^37^.

Inflammation is the main result of oxidative stress, that activates the release of active NF-κB. NF-κB, itself, is a redox sensitive transcription factor that regulate inflammation through triggering the transcriptional upregulation of inflammatory COX-2 and some pro-inflammatory cytokines, such as IL-6 and TNF-*α*. NF-κB and COX-2 are the important enzymes of inflammatory signaling pathways^4^. The present data exhibited a possible anti-inflammatory activity of both extracts by attenuating the highly expressed immunostaining level of COX-2 and gene expression of NF-κB in B(a)P-treated group. Recinella and co-workers^38^, supported our results by presenting *Vitis vinifera* L. leaves extract as potential anti-inflammatory extract through alleviating both COX-2 and NF-κB in isolated mouse colon.

Nitric oxide (NO) is a free radical that can break DNA strands. By reacting with oxygen and superoxide anion, NO forms the cytotoxic radicals called nitrogen dioxide and peroxynitrite which have a bad influence on the lung functional parameters^39,40^. The inducible nitric oxide synthase (iNOS) is highly expressed in inflammatory and neoplastic conditions^40^. NF-κB activation was proven to increase the expression of iNOS along with COX-2 enzyme^41^. In the present study, VLME extracts was found to be effectively modulating the over expression of iNOS by [B(a)P] which presents an additional approval for VLME to protect against chemical-induced lung insult. Earlier study conducted by^42^, revealed similar findings, where *Vitis vinifera* suppressed COX-2 and iNOS expression in CCl_4_ induced multiorgan toxicity in rats.

A member of the CD34 protein family, CD34 is a broadly expressed sialomucin that selectively glycosylates in the high endothelial venules. It has been shown to play an essential role in the recruitment of leukocytes in various allergic diseases^43^. It has been shown that activation of neutrophils is a crucial stage in the organ’s defensive system when recruiting them into inflammatory tissues, but also these over activated cells may cause excessive tissue damage. Also, positive expressed monocyte was revealed to enhance T cell activation through iNOS^43^. Herein, our data showed that mice group received [B(a)P] expressed high CD34 score, while VLME extracts provided a gradual decrease in the CD34 immunoscoring, on a dose dependent manner.

Caspase 3 is one of the main controllers of apoptosis, which mediate cell death. Our results exhibited a significant decrease of caspase 3 expression in VLME pretreated groups, compared to the positive control group. Our findings were confirmatory to those reported for CCl_4_-induced liver damage that was ameliorated by *Vitis vinifera* L. leaves extract through decreasing apoptotic caspase 3 level^44^. The histological observations presented herein verified the preceding findings, in which [B(a)P] had a destructive and inflammatory effect on lung alveoli and tissue structure, whereas pretreatment with VLME improved lung tissues by ameliorating congestion and inflammation signs.

## Conclusion

The LC-ESI-MS/MS investigation of VLME tentatively recognized 52 and 47 different compounds in negative and positive ESI modes, respectively. The major constituents were quercetin-3-*O*-glucuronide, kaempferol-3-*O*-glucoside, choline, *γ*-linolenic acid, 3,3′,4′,5-tetrahydroxy-7-methoxyflavone, hyperoside, 1-*O-β*-D-glucopyranosyl sinapate, and quercetin. Taraxerol **(1)** and quercetin-3-*O*-*β*-D-glucuronoide − 6″-methyl ester **(4)** had first isolated from *V. vinifera*. The histopathological and immunohistochemical improvements by the plants’ extracts as well as the downregulation effect on MDA serum level and gene expression of caspase 3, NF-ҡB may explore the underlying mechanisms of the potential lung protective effect. Building upon the results and findings from the pre-clinical in vivo studies on mice, further clinical investigations will be recommended.

## Materials and methods

**Instruments and reagents**: **Supplementary Materials**.

### Material for biological study

The solvents utilized were of HPLC grade > 99.9% and acquired from Sigma Co. (St. Louis, MO, USA). Benzo(a)pyrene [B(a)P], was purchased from Sigma-Aldrich, MO, USA. Colorimetric kits for MDA and GSH measurement were obtained from Biodiagnostics, Egypt. Antibodies for COX2, CD34 and iNOS were purchased from (ABclonal Technology, Woburn, MA, USA).

### Plant material

Leaves of *Vitis vinifera* L. var. King Ruby were collected from garden in Kotor Tanta road, in August 2020. King Ruby variety was identified by Prof. Dr. Osama Kamal El-Abasy, Professor of Horticulture, Faculty of Agriculture and Prof. Dr. Nabil El-Sheery, Professor of Agriculture Botany, Faculty of Agriculture. Voucher samples were stored at the herbarium of the Pharmacognosy Department, Faculty of Pharmacy, Tanta University with No. PGA-8-1-2020.

**Extraction and isolation**: **Supplementary Materials**.

### LC-ESI-MS/MS of VLME

The Proteomics and Metabolomics Unit, Basic Research Department, Children’s Cancer Hospital (57357), Cairo, Egypt, is where chemical identification of compounds was tentatively done. Using the methods already described in the literature^45^.

### In vitro anticancer impact of VLME: (SRB Assay)

Nawah Scientific Inc. (Mokatam, Cairo, Egypt) supplied A-549 (lung cancer) cell line. Humidified with 5% CO_2_ at 37 °C. The SRB cell viability test was done. 100 µL cell suspension (5 × 10^3^ cells) were cultivated in 96-well plates for 24 h. Various concentration (0.03, 0.3, 3, 30, 300 µg/mL) of VLME var. King ruby (four fractions) were added to 100 µL medium and applied to cells. After 72 h of drug treatment, the cells were fixed by adding 150 µL of 10% trichloroacetic acid (TCA) to the medium and incubating for one hour in the refrigerator. The TCA solution was removed from the equation, and cells were rinsed five times with distilled.

### In vivo lung protective effect of VLME

#### Experimental animals

The National Research Center in Giza, Egypt, provided sixty male 20–25 g Swiss Albino mice. For 14 days, all of the mice were allowed to acclimate and were given access to a typical rodent feed and clean tap water. All mice were kept in cages with a 12-hour light-dark cycle, 24 ± 2 °C, and 60–70% relative humidity.

### Ethics statement

The animal handling was stuck to the approved guidelines of the research ethics committee for the care and use of laboratory animals, at Faculty of Pharmacy, Tanta University (approval number of TP/RE/3/24 p-03). In terms of ethical standards, the Egyptian Guide for the Care and Use of Laboratory Animals has been followed. The local ethics committee accepted the experimental protocol, which fully adhered to the ARRIVE criteria for reporting in vivo investigations and employing laboratory animals. The animals were killed via cervical dislocation following the administration of ketamine (50 mg/kg i.p.) and xylazine (10 mg/kg i.p.) for anesthesia^46,47^.

#### Experimental protocol

##### Mice were randomly allocated into 10 groups (*n* = 6) as follows

**Group I** (negative or normal control, mice received saline), **group II** (disease control, mice received B(a)P 125 mg/kg, orally), **group III** (mice received (VLME)100 mg/kg, orally, followed by B(a)P), **group IV** (mice received (VLME) 200 mg/kg, orally followed by B(a)P), **group V** (mice received (VLME) 300 mg/kg, orally, followed by B(a)P) and **group VI** (mice received only (VLME) 300 mg/kg, orally, only), **group VII** (mice received (MCF) 100 mg/kg, orally, followed by B(a)P), **group VIII** (mice received (MCF) 200 mg/kg, orally, followed by B(a)P), g**roup IX** (mice received (MCF) 300 mg/kg, orally, followed by B(a)P), **group X** (mice received (MCF) 300 mg/kg, orally, only).

Groups **I** and **II** were given saline for seven consecutive days, while groups **III**,** IV**,** V** and **VI**,** VII**,** VIII**,** IX**,** X** were given the extract orally for seven days. On the seventh day, all groups received B(a)P), mice received a single oral dose of B(a)P 125 mg/kg body weight dissolved in corn oil via an oral gavage tube^48,49^, except group **I**,** VI and X**. After 24 h of receiving B(a)P or saline (for group I) all mice were euthanized, and the lungs of each was excised at necropsy; A small portion of each was kept in 10% formalin solution for histological and immunohistochemical evaluation and the remaining was kept in -80 °C for further biochemical measurements.

#### Colorimetric determination of MDA and GSH levels

Phosphate-buffered saline (PBS, pH 7.5) was used for tissue homogenization, using homogenizer (Polytron, PT 3100, Switzerland) followed by centrifugation at 4 °C.

(4000 rpm for 15 min). After that, the samples were handeled following the manufacturer protocol. The MDA and GSH levels were measured at wavelengths of 450 nm and 534 nm by UV-VIS spectrophotometer (Unico, NJ, USA), using the following equations, respectively:

1 $$\:MDA\:level\:(nmol/g\:tissues)=\frac{A\:sample}{A\:standard}\times\:\frac{10}{weight\:of\:tissues\:used\:\left(g\right)}$$

2 $$\:GSH\:concentration\:(mg/g\:tissues)=\frac{A\:sample\:\times\:66.66}{weight\:of\:tissues\:used\:\left(g\right)}$$

#### Determination of Caspase 3 and NF-ҡB levels

qRT-PCR was used to assess the relative gene expression of NF-кB and caspase 3 using GAPDH as a housekeeping gene. The extraction step of the total RNA was conducted using TRIzol reagent (15596026) (Life Technologies, 170 USA). The reverse transcription step was performed applying QuantiTects Reverse transcription kit (Qiagen, USA). The reaction mixtures including primers, complementary DNA amplicons and Syber green master mix (Maxima SYBR Green/qPCR Master Mix, Thermo Fisher Scientific, USA) were used for the last step. To compute the fold change in gene expression, the Livak method was applied^50^. The primers sequences were listed in Supplementary Materials, Table S1.

#### Histopathological studies

Lung tissues were embedded in paraffin blocks after being fixed in 10% formalin for histological examination. Using a Leica RM2135 microtome (Leica, Berlin, Germany), the samples were sectioned serially to a thickness of 5 μm, then placed onto glass slides, and stained with H&E solution. A light microscope was used to examine these sections^51^.

#### Immunohistochemical studies

Sections of lung tissue were sliced from the paraffin-embedded tissue blocks and placed on poly lysine slides. After the sections were dewaxed, dried, and rehydrated, they were incubated in H_2_O_2_, cleaned in PBS, and boiled in citrate buffer. Antibody staining was then applied to the sections.

Quantification was performed according to the following semiquantitative scores for [COX-2, CD34 and iNOS] based on the percentage of cytoplasmic positively stained cells (brownish in color) of the alveolar, bronchial epithelium and inflammatory cells, the scoring: 0, no staining; 1 weak, ≤ 25%; 2 moderate, 26–50%; 3 strong, 51–75%; and 4 marked, 76–100%^52^.

#### Statistical analysis

Data analysis was performed using Graphpad Prism 8 (San Diego, USA). Using one-way analysis of variance (ANOVA), the difference between groups was measured. The findings were plotted as mean ± standard deviation (SD). The *p*-values less than 0.05 were considered statistically significant.

## Electronic supplementary material

Below is the link to the electronic supplementary material.

Supplementary Material

## Data Availability

All data generated or analysed during this study are included in this published article [and its supplementary information files].
