# Supplementary material for: Phytochemicals of Vitis vinifera L. var. King Ruby protect mice from benzo(a)pyrene-induced lung injury
Source: Sci Rep. 2025 Feb 6;15:4536. doi: 10.1038/s41598-025-86173-x (PMC11802910; doi:10.1038/s41598-025-86173-x)
Supplement: Supplementary file 1 — Supplementary Material [file 41598_2025_86173_MOESM1_ESM.docx]

**Phytochemicals of** ***Vitis vinifera* L. var. King Ruby Protect Mice from Benzo(a)Pyrene-Induced Lung Injury**

Gehad S. Ahmedy^1^, Hend M. Selim^2^, Mona El-Aasr^1*^, Souzan M. Ibrahim^1^, Suzy A. El-Sherbeni^1*^

^1^ Department of Pharmacognosy, Faculty of Pharmacy, Tanta University, Tanta 31527, Egypt.

^2^ Department of Biochemistry, Faculty of Pharmacy, Tanta University, Tanta 31527, Egypt.

^*^ Corresponding authors: [moelaasar@pharm.tanta.edu.eg](mailto:moelaasar@pharm.tanta.edu.eg)

suzy.elsherbini@pharm.tanta.edu.eg

| **TABLE OF CONTENTS** | |
| --- | --- |
| **Subject** | **Page** |
| **Figure SL1:** TIC of *Vitis vinifera* L. leaves methanolic extract (King Ruby variety) [Negative mode] | 3 |
| **Figure SL2:** TIC of *Vitis vinifera* L. leaves methanolic extract (King Ruby variety) [Positive mode] | 4 |
| **Figure SL3:** Major compounds in LC-ESI-MS/MS | 5 |
| Instruments and reagents | 6 |
| Extraction and isolation of compounds | 6 |
| Characterization of the isolated compounds | 8 |
| Discussion about the structure elucidation of the isolated compounds | 10 |
| **Scheme S1:** Extraction and fractionation steps of *Vitis vinifera* L. var. King Ruby leaves | 13 |
| **Scheme S2:** Investigation of PE fraction of *Vitis vinifera* L. var. King Ruby leaves | 14 |
| **Scheme S3:** Column chromatography of USM residue of *Vitis vinifera* L. var. King Ruby leaves | 15 |
| **Scheme S4:** Column chromatography of MCF residue of *Vitis vinifera* L. var. King Ruby leaves | 16 |
| **Scheme S5:** Column chromatography of EtOAc residue of *Vitis vinifera* L. var. King Ruby leaves | 17 |
| **Figure S1A:** IR spectrum of compound **1** [KBr disc] | 18 |
| **Figure S1B:** EI-MS spectrum of compound **1** | 18 |
| **Figure S1C:** ^1^H-NMR spectrum of compound **1** [CDCl_3_, 400 MHz] | 19 |
| **Figure S1D:** ^13^C-NMR spectrum of compound **1** [CDCl_3_, 100 MHz] | 19 |
| **Figure S2A:** IR spectrum of compound **2** [KBr disc] | 20 |
| **Figure S2B:** EI-MS spectrum of compound **2** | 20 |
| **Figure S2C:** ^1^H-NMR spectrum of compound **2** [CDCl_3_, 400 MHz] | 21 |
| **Figure S2D:** ^13^C-NMR spectrum of compound **2** [CDCl_3_, 100 MHz] | 21 |
| **Figure S3A:** IR spectrum of compound **3** [KBr disc] | 22 |
| **Figure S3B:** EI-MS spectrum of compound **3** | 22 |
| **Figure S3C:** ^1^H-NMR spectrum of compound **3** [DMSO-*d_3_*, 400 MHz] | 23 |
| **Figure S3D:** DEPTQ-NMR spectrum of compound **3** [DMSO-*d_3_*, 100 MHz] | 23 |
| **Figure S4A:** UV spectrum of compound **4** | 24 |
| **Figure S4B:** IR spectrum of compound **4** [KBr disc] | 25 |
| **Figure S4C:** ESI-MS spectrum of compound **4** [Negative mode] | 25 |
| **Figure S4D:** ^1^H-NMR spectrum of compound **4** [CD_3_OD, 400 MHz] | 26 |
| **Figure S4E:** DEPTQ-NMR spectrum of compound **4** [CD_3_OD, 100 MHz] | 26 |
| **Figure S4F:** HMBC spectrum of compound **4** | 27 |
| **Figure S5A:** UV spectrum of compound **5** | 28 |
| **Figure S5B:** IR spectrum of compound **5** [KBr disc] | 29 |
| **Figure S5C:** ESI-MS spectrum of compound **5** [Negative mode] | 29 |
| **Figure S5D:** ^1^H-NMR spectrum of compound **5** [CD_3_OD, 400 MHz] | 30 |
| **Figure S5E:** APT-NMR spectrum of compound **5** [CD_3_OD, 100 MHz] | 30 |
| **Figure S5F:** HMBC spectrum of compound **5** | 31 |
| **Figure S1:** Impact of different pre-treatments on (A) MDA and (B) GSH levels. Data are revealed as mean ± SD, n=6. a: reflects a significant change (p<0.05) against the normal control, b: reflects a significant change (p<0.05) against the positive control group. | 32 |
| **Figure S2:** Impact of different pre-treatments on (A) Caspase 3 and (B) NF-ҡB gene expression. Data are revealed as mean±SD, n=6 a: reflects a significant change (p<0.05) against the normal control, b: reflects a significant change (p<0.05) against the positive control group. | 32 |
| **Table S1:** Primer sequences of Caspase 3 and NF-кB | 32 |
| **Supplementary Table SL1:** Phytochemical profiling of VLME by LC-ESI-MS/MS analysis (Negative ESI mode): | 33 |
| **Supplementary Table SL*2*:** Phytochemical profiling of VLME by LC-ESI-MS/MS analysis (Positive ESI mode): | 38 |
| References | 42 |

**Figure SL1:** TIC of *Vitis vinifera* L. var. King Ruby leaves methanolic extract [Negative mode]


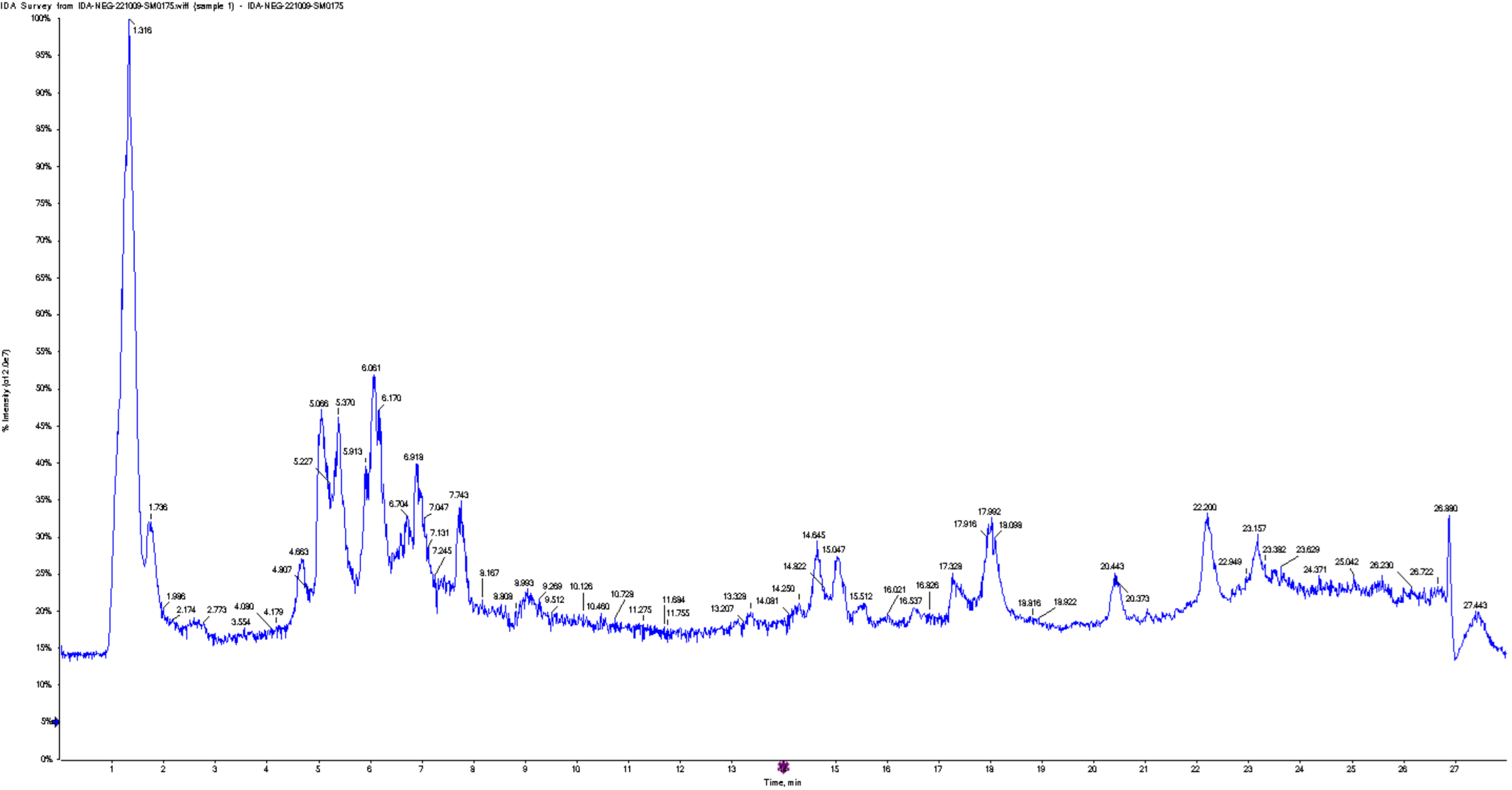


**Figure SL2:** TIC of Vitis vinifera L. var. King Ruby leaves methanolic extract [Positive mode]


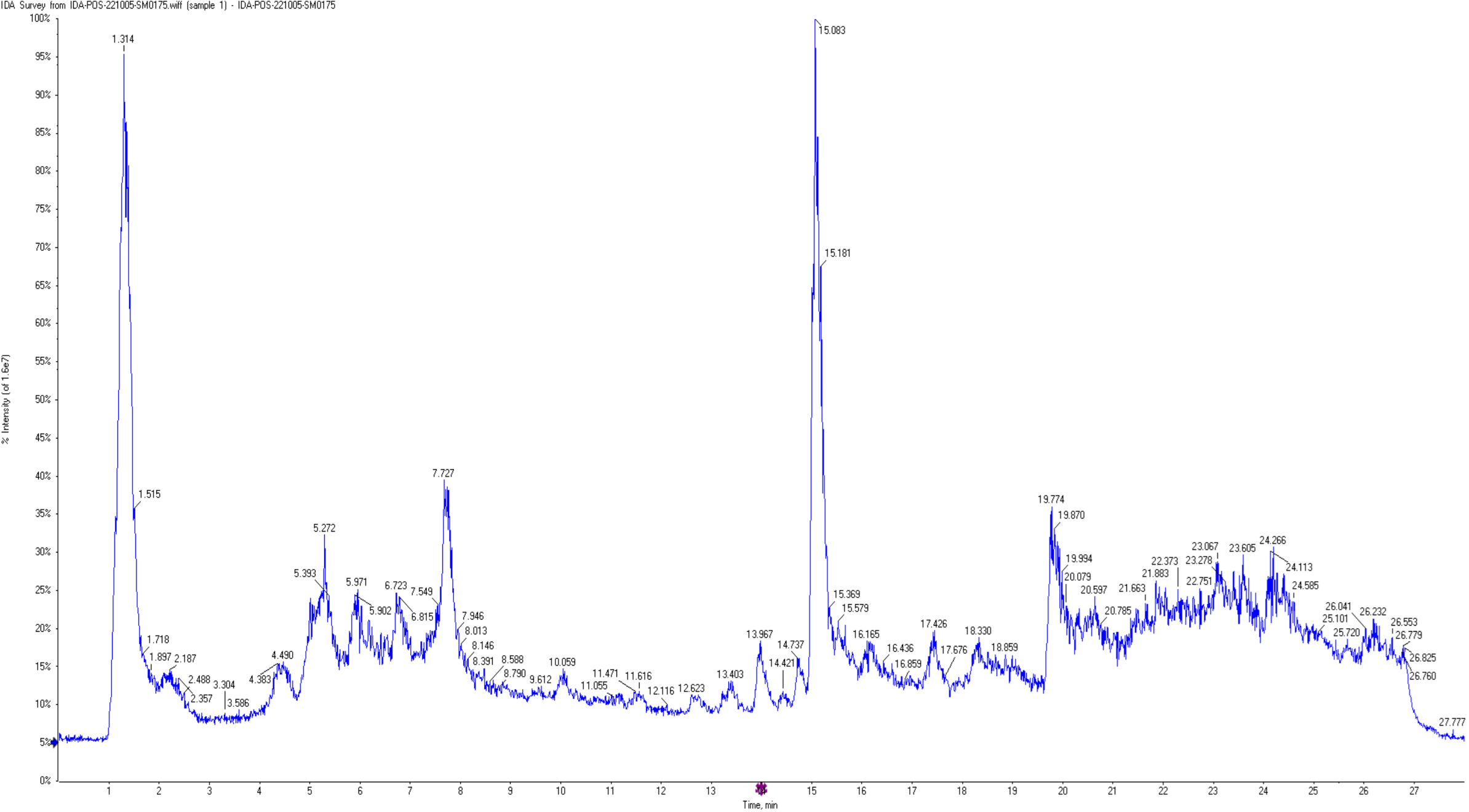


**Figure SL3: Major compounds in LC-ESI-MS/MS**

| Quercetin | Hyperoside |
| --- | --- |
|  |  |
| 3, 3՛, 4՛, 5-tetrahydroxy-7-methoxyflavone | Kaempferol-3-*O*-glucoside |
|  |  |
| Quercetin-3-*O*-glucuronide | 1-*O*-*β*-D-glucopyranosyl sinapate |
|  |  |
| Choline | γ-Linolenic acid |
|  |  |

# **Instruments and reagents**

1. **Instruments:**

NMR investigations were carried out with a Bruker Avance III spectrometer (Germany) at 400 MHz for ^1^H and 100 MHz for ^13^C-NMR, APT and DEPT-Q NMR. The UV spectra were collected using a Shimadzu UV/VIS spectrophotometer UV-1800 (Tokyo, Japan). Jasco Co.'s FT/IR-6100 spectrophotometer (Tokyo, Japan) was used to analyze IR spectra of KBr discs. ESI-MS spectra were obtained using a compact mass spectrometer (CMS) from Advion Co. (New York, NY, USA) while EI-MS spectra were obtained using Direct Inlet part to mass analyzer in Thermo Scientific GCMS model ISQ at the Regional Center for Mycology and Biotechnology (RCMB), Al-Azhar University, Nasr City, Cairo.

1. **Reagents and solvents:**

The solvents employed for extraction and fractionation were of analytical grade, while for Sephadex, HPLC methanol was used. Sephadex LH-20 was provided from Sigma-Aldrich Chemical Co. (St. Louis, MO, USA), Silica gel (70–230 mesh) and precoated TLC sheets of silica gel F254 were purchased from Merck Co. (Darmstadt, Germany). Authentic sample of glucose for Co-TLC was provided by the Department of Pharmacognosy, Faculty of Pharmacy, Tanta University, Egypt. TLC detection was carried out using spray reagents AlCl_3_ (5%) and H_2_SO_4_ (10%) and the solvent systems used for TLC were **S1:** *n*-Hexane-EtOAc (8.5:1.5), **S2:** CH_2_Cl_2_-MeOH (9:1), **S3:** CH_2_Cl_2_-MeOH-H_2_O (9:1:0.1), **S4:** CH_2_Cl_2_ - MeOH (8.5:1.5), **S5:** CH_2_Cl_2_-MeOH-H_2_O (8:2:0.2), **S6:** CH_2_Cl_2_-MeOH-H_2_O (7:3:0.5), **S7:** CH_2_Cl_2_-MeOH-H_2_O (6:4:0.5) and **S8:** *n*-Butanol - Acetic acid - Water (BAW) (4:1:5).

# **Extraction and isolation of compounds:**

The dried powder (3 kg) was extracted by cold maceration method using methanol (3 × 10 L, 72 h each) till exhaustion at room temperature. Then concentrated under reduced pressure to yield 400 g residue of total methanol extract (VLME). 200 gram of VLME were fractionated using four solvents (Petroleum ether 60-80 ˚C (PE), methylene chloride (MCF), ethyl acetate (EtOAc) and finally *n*- butanol (*n-*BuOH) till exhaustion to afford four fractions, and they are PE (45.6 g), MCF (9.5 g), EtOAc (6.8 g) and *n*-BuOH (24.8 g) fractions **(Scheme S1)**.

1. **Petroleum ether fraction (PE)** (45.6 g) was used to investigate the saponifiable and unsaponifiable matter. 12 g residue from the pet-ether (60–80 °C) fraction was saponified by heating 180 mL (10%) alcoholic KOH on a boiling W.B. with an air condenser for 6 hours. Following the distillation of the alcohol from the saponified mixture, the remaining liquid was mixed with 120 mL of water and repeatedly extracted using diethyl ether until it was exhausted. To get the (USM) of King Ruby leaves, the combined ethereal extracts were washed with D.W. then dehydrated over anhydrous Na_2_SO_4_ and distilled out under low pressure **(Scheme S2)**. 6 g residue of the USM residue **(Scheme S2)** was chromatographed on a silica gel column (⌀ 3 × 35 cm, 180 g silica gel). Gradient elution was used, beginning with pet. ether and increasing polarity by 10% using CH_2_Cl_2_. Fractions (50 ml each) were collected and screened by TLC on silica gel sheets using solvent system S1, S2, S3, S4 and 10% H_2_SO_4_ reagent is used for detection to result in 4 groups P-1 (1 red major and 2 brown minor spots), P-2 (2 minor brown spots), P-3 (1 major purple and 1 minor brown spots), and P-4 (2 minor black spot). The P-1 group (1211 mg) was then chromatographed on a silica gel column (⌀ 1.5 × 50 cm, 36 g silica gel) by dry packing and gradient elution starting with pet. ether:CH_2_Cl_2_ (90:10) and the polarity was increased by 5% using CH_2_Cl_2_ and collecting fractions (10 ml each) then exanimated by TLC on silica gel sheets using solvent system S1, S2, S3 and 10% H_2_SO_4_ for detection to afford 5 subgroups (P-1-A to P-1-E). Subgroup P-1-C (232 mg) with a major red spot was dissolved in pyridine and MeOH to isolate **Compound 1** (50 mg) using Sephadex LH-20 column (⌀ 1.5 × 25 cm, 20 g) and 100% MeOH. On a silica gel column (⌀ 1.5 × 23 cm, 15 g silica gel), P-3 group (452 mg) was chromatographed by dry packing and gradient elution method starting with pet. ether:CH_2_Cl_2_ (80:20) and the polarity was increased by 5% using CH_2_Cl_2_ and collecting fractions (10 ml each) then exanimated by TLC on silica gel sheets using solvent system S1, S2, S3 and 10% H_2_SO_4_ for detection to give 5 subgroups (P-3-A to P-3-E). Subgroup P-3-E (201 mg) had a major purple spot and needle shaped crystals. So, it was dissolved in pyridine and MeOH to isolate **Compound 2** (30 mg) using Sephadex LH-20 column (⌀ 1.5 × 25 cm, 20 g) and 100% MeOH, followed by repeated crystallization with MeOH **(Scheme S3)**.
2. **Methylene chloride fraction (MCF)** (5 g) was chromatographed on a silica gel column (⌀ 3 × 32 cm, 150 g silica gel). Gradient elution was used, beginning with *n.* hexane and increasing polarity using CH_2_Cl_2_ till 100 % CH_2_Cl_2_ then MeOH is used by 1% increase till CH_2_Cl_2_:MeOH (90:10). Fractions (50 ml each) were collected and examined by TLC on silica gel sheets using solvent system S1, S2, S3 and 10% H_2_SO_4_ for detection to result in 3 groups C-1 (1 major green and 2 minor brown spots), C-2 (1 major green and 1 minor brown spots), and C-3 (1 major red spot) **(Scheme S4)**. **Compound 3** (35 mg) was isolated from group C-3 (419 mg) after chromatography on a silica gel column (⌀ 1.5 × 22 cm, 13 g silica gel) by wet packing and gradient elution method starting with 100% CH_2_Cl_2_ the polarity was increased by 1% using MeOH, followed by collecting fractions (10 ml each) and examined by TLC on silica gel sheets using solvent system S1, S2, S3 and 10% H_2_SO_4_ for detection to give 3 subgroups (C-3-A to C-3-C) then crystallization was performed on C-3-B subgroup (50 mg) as it had the major red spot.
3. **Ethyl acetate** **fraction (EtOAc)** (4 g) was chromatographed on a silica gel column (⌀ 3 × 29 cm, 120 g silica gel). Gradient elution was used, beginning with CH_2_Cl_2_ and increasing polarity using MeOH by 5%. Fractions (50 ml each) were collected and screened by TLC on silica gel sheets using solvent system S5, S6 and S7 while 5% AlCl_3_ reagent was used for detection result in 5 groups E-1 (1 minor yellow spot), E-2 (3 minor brown spots), E-3 (1 major yellow and 1 minor brown spots), E-4 (1 major yellow spot), and E-5 (1 major yellow spot) **(Scheme S5)**. The E-3 group (1479 mg) was chromatographed on a silica gel column (⌀ 2.5 × 30 cm, 45 g silica gel) by dry packing and gradient elution method starting with 100% CH_2_Cl_2_ and the polarity was increased by 1% using MeOH. Fractions (10 ml each) were collected and examined by TLC on silica gel sheets using solvent system S5, S6, S7 and 5% AlCl_3_ reagent was used for detection to give 5 subgroups (E-3-A to E-3-E). Using Sephadex LH-20 column (⌀ 1.5 × 25 cm, 20 g) and 100% MeOH, **Compound 4** (55 mg) was isolated from subgroup E-3-C (320 mg) which had yellow powder with major yellow spot on TLC. On a silica gel column (⌀ 1.5 × 24 cm, 17 g silica gel) E-5 (570 mg) group was chromatographed by dry packing and gradient elution method in which started with 100% CH_2_Cl_2_ and the polarity was increased by 1% using MeOH. Fractions (10 ml each) were collected and examined by TLC on silica gel sheets using solvent system S4, S5, S6 and 5% AlCl_3_ reagent was used for detection to give 4 subgroups (E-5-A to E-5-D). Using Sephadex LH-20 column (⌀ 1.5 × 25 cm, 20 g) and 100% MeOH, **Compound 5** (30 mg) was isolated from subgroup E-5-C which had major yellow spot on TLC.

**Acid hydrolysis**

By heating 2 mg of each isolated glycoside (isoquercetin and daucosterol) with 5 mL of methanol and air refluxing with 2 N HCl at 100 °C in a water bath with an air condenser for two hours, acid hydrolysis was achieved. The resulting mixture was neutralized with NaOH after it had evaporated. The neutralized product was analyzed by TLC and compared with authentic samples using S8 as solvent system ^1^.

# **Characterization of the isolated compounds**

**Compound 1:**

White amorphous powder (50 mg) with R*_f_* = 0.85 using S3 and after heating and spraying with 10% H_2_SO_4_ reagent, it leaves a red spot on TLC.

It is soluble in chloroform with heating, sparingly soluble in methanol with m.p. (276 °C), The results of the chemical testing for sterols and/or triterpene by Salkowski’s and Liebermann were positive.

**IR** spectrum **Figure S1A** showed a broad absorption band at 3450.10, 2924.28, 2854.78 and 1636.13 cm^-1^ vibrations.

**EI-MS** spectrum **Figure S1B** showed molecular ion peak at *m/z* 426.38 [M]^+^ with peaks 411, 302, 269, 204,189.

**^1^H-NMR** spectrum **Figure S1C** [in CDCl_3_, 400 MHz] δ: 5.55 (1H, dd, J = 3.16, 8.1 Hz, H-15), 3.23 (1H, m, H-3), 1.27 (3H, s, H-27), 1.11 (3H, s, H-23), 1.00 (3H, s, H-29), 0.97 (3H, s, H-24), 0.95 (3H, s, H-26), 0.93 (3H, s, H-30), 0.84 (3H, s, H-28) and 0.82 (3H, s, H-25).

**^13^C-NMR** spectrum **Figure S1D** [CDCl_3_, 100 MHz] δ: 158.10 (C-14), 116.80 (C-15), 79.08 (C-3), 55.55 (C-5), 49.30 (C-18), 48.78 (C-9), 41.34 (C-19), 38.99 (C-4), 38.76 (C-8), 38.01 (C-1), 37.75 (C-10), 37.72 (C-17), 37.58 (C-13), 36.68 (C-16), 35.78 (C-12), 35.13 (C-7), 33.71 (C-21), 33.35 (C-29), 33.11 (C-22), 29.91 (C-26), 29.83 (C-28), 29.69 (C-20), 28.00 (C-23), 27.16 (C-2), 25.90 (C-27), 21.31 (C-30), 18.80 (C-6), 17.50 (C-11), 15.44 (C-24), and 15.42 (C-25).

**Compound 2:**

White needle crystals (30 mg) with R*_f_* = 0.55 using S3 and after heating and spraying with 10% H_2_SO_4_ reagent, it leaves a purple spot-on TLC.

It is soluble in ether and chloroform, sparingly soluble in methanol with m.p (139 °C). The results of the chemical testing for sterols and/or triterpene by Salkowski’s and Liebermann were positive.

**IR** spectrum **Figure S2A** showed a broad absorption band at 3475.76, 2923.19, 2853.78 and 1619.04 cm^-1^ vibrations.

**EI-MS** spectrum **Figure S2B** showed molecular ion peak at *m/z* 414.58 [M]^+^ with peaks at *m/z* 396, 381, 273 and 255.

**^1^H-NMR** spectrum **Figure S2C** [CDCl_3_, 400 MHz] δ: 5.28 (1H, d, J = 5.4 Hz, H-6), 3.45 (1H, m, H-3), 0.94 (3H, s, H-19), 0.90 (3H, t, J = 6.7 Hz, H-29), 0.85 (3H, d, J = 6.4 Hz, H-21), 0.78 (3H, d, J=7.76 Hz, H-26), 0.74 (3H, d, J = 6.84 Hz, H-27), 0.61 (3H, s, H-18) and 0.63-2.21 (m, H-1, 2, 4, 7-9, 11, 12, 14-17, 20, 22-25, 28).

**^13^C-NMR** spectrum **Figure S2D** [CDCl_3_, 100 MHz] δ: 140.78 (C-5), 121.72 (C-6), 71.82 (C-3), 56.79 (C-14), 56.09 *(*C-17), 50.16 (C-9), 45.87 (C-24), 42.34 (C-4 and C-13), 39.80 (C-12), 37.27 (C-1), 36.52 (C-10), 36.15 (C-20), 33.97 (C-22), 31.93 (C-7), 31.69 (C-8), 29.69 (C-2), 29.19 (C-25), 28.25 (C-16), 26.13 (C-23), 24.31 (C-15), 23.09 (C-28) 21.10 (C-11), 19.81 (C-27), 19.40 (C-19), 19.05 (C-21), 18.79 (C-26), 11.99 (C-18) and 11.86 (C-29).

**Compound 3:**

White amorphous powder (35 mg) with R*_f_* = 0.62 using S4 and after heating and spraying with 10% H_2_SO_4_ reagent, it leaves a purple spot on TLC.

It is soluble in chloroform with heating, sparingly soluble in methanol with m.p. (283 °C), The results of the chemical testing for sterols and/or triterpene by Salkowski’s and Liebermann were positive and gave positive Molisch’s test but did not reduce Fehling’s solution.

**IR** spectra **Figure S3A** showed peaks at 3449.48, 2854.19, 2923.79 and 1636.05 cm^-1^.

**ESI-MS** mass spectrum **Figure S3B** of compound **3** displayed peak at *m/z* 575.4.

**^1^H-NMR** spectrum **Figure S3C** [DMSO-*d_6_*, 400 MHz] δ: 5.34 (1H, br s, H-6), 4.23 (1H, d, J = 7.8 Hz, H-1՛), 3.65 (1H, m, H-3), 0.96 (3H, s, H-19), 0.91 (3H, d, J = 6.4 Hz, H- 21), 0.84 (3H, d, J = 6.76 Hz, H-26), 0.82 (3H, t, J = 7.08 Hz, H-29), 0.80 (3H, d, J = 7.12 Hz, H-27), 0.66 (3H, s, H-18) and 0.66- 2.14 (m, H-1, 2, 4, 7-9, 11, 12, 14-17, 20, 22-25, 28) and 2.91-4.87 (m, Glucose protons).

**^13^C-NMR** spectrum **Figure S3D** [DMSO-*d_6_*, 100 MHz] δ: 140.95 (C-5), 121.69 (C-6), 77.78 (C-3), 56.65 (C-14), 55.90 (C-17), 50.08 (C-9), 45.62 (C-24), 42.33 (C-13), 39.37 (C-4), 38.78 (C-12), 37.31 (C-1), 36.71 (C-10), 35.95 (C-20), 33.82 (C-22), 31.91 (C-8), 31.84 (C-7), 29.74 (C- 2), 29.19 (C-25), 28.26 (C-16), ), 25.92 (C-23), 24.33 (C-15), 23.08 (C-28), 21.06 (C-11), 20.19 (C-26), 19.58 (C-27), 19.42 (C-19), 19.09 (C-21), 12.26 (C-29). 12.15 (C-18), Glucose moiety δ:101.26 (C-1՛), 77.39 (C-3՛) 77.25 (C-5՛), 73.95 (C-2՛), 70.60 (C-4՛) and 61.59 (C-6՛).

**Compound 4:**

Yellow amorphous powder (55 mg) with R*_f_* = 0.68 using S5 and give yellow spot after spraying with AlCl_3_ solution on TLC.

It is soluble in methanol and acetone. with m.p (318 °C). The result of the Molisch’s test was positive which indicate presence of flavonoid glycoside.

**UV** λmax at 243, 261 and 300 nm **Figure S4A**.

**IR** spectrum **Figure S4B** showed a broad absorption band at 3416.02 cm^-1^ for O-H stretching vibration in addition to absorptions at 1619.37 cm^-1^ for a carbonyl (C=O) group.

**ESI-MS** spectrum **Figure S4C** negative mode mass spectrum of compound **4** displayed peaks at *m/z* 491.3 and 537.3.

**^1^H-NMR** spectrum **Figure S4D** [400 MHz, CD_3_OD] δ: 7.49 (1H, dd, J = 2.4, 9.0 Hz, H-6՛), 7.48 (1H, d, J = 2.4 Hz, H-2՛), 6.74 (1H, d, J = 8.8 Hz, H-5՛), 6.30 (1H, d, J = 2.0 Hz, H-8), 6.11 (1H, d, J = 2.0 Hz, H-6), 5.14 (1H, d, J = 7.6 Hz, H-1՛՛), 3.65 (1H, d, J = 9.6 Hz, H-5՛՛), 3.55 (3H, s, 6՛՛ OCH_3_) and 3.20-3.48 (3H, m, H-2՛՛, 3՛՛, 4՛՛).

**^13^C-NMR** spectrum **Figure S4E** [100 MHz, CD_3_OD] δ: 177.83 (C-4), 164.83 (C-7), 161.65 (C-5), 157.97 (C-2), 157.08 (C-9), 148.49 (C-4՛), 144.57 (C-3՛), 134.01 (C-3), 122.06 (C-6՛), 121.47 (C-1՛), 116.23 (C-2՛), 114.48 (C-5՛), 104.17 (C-10), 98.59 (C-6), 93.40 (C-8).

Glucuronide moiety δ: 169.26 (C-6՛՛), 103.32 (C-1՛՛), 75.91 (C-5՛՛), 75.70 (C-3՛՛), 73.51 (C-2՛՛), 71.33 (C-4՛՛) and 6՛՛ methyl ester δ: 51.44.

**Compound 5:**

Yellow amorphous powder (30 mg) with R*_f_* = 0.52 using S5 and give yellow spot after spraying with AlCl_3_ solution on TLC.

It is soluble in methanol and acetone. with m.p (230 ^o^C). The result of the Molisch’s test was positive which indicates presence of flavonoid glycoside.

**UV** λmax at 243, 253, 313 and 337 nm **Figure S5A**

**IR** spectrum **Figure S5B** showed a broad absorption band at 3450.10 cm^-1^ for O-H stretching vibration in addition to absorptions at 1638.59 cm^-1^ for a carbonyl (C=O) group.

**ESI-MS** spectrum **Figure S5C** negative mode mass spectrum of compound **5** displayed peaks at *m/z* 463.3.

**^1^H-NMR** spectrum **Figure S5D** [400 MHz, CD_3_OD] δ: 7.61 (1H, d, J = 2.2 Hz, H-2՛), 7.49 (1H, dd, J = 2.2, 8.56 Hz, H-6՛), 6.77 (1H, d, J = 8.48 Hz,H-5՛), 6.30 (1H, d, J = 2.1 Hz, H-8), 6.11 (1H, d, J = 2.1 Hz, H-6), 5.15 (1H, d, J = 7.52 Hz, H-1՛՛), 3.61 (1H, dd, J = 11.9, 2.4 Hz, H-6՛՛*α*), 3.47 (1H, dd, J = 11.9, 5.28 Hz, H-6՛՛*β*), 3.40 (1H, d, J = 8.92 Hz, H- 2՛՛), 3.36 (1H, d, J = 7.28 Hz, H-3՛՛), 3.32 (1H, d, J = 9.0 Hz, H-4՛՛) and 3.14 (1H, m, H- 5՛՛).

**^13^C-NMR** spectrum **Figure S5E** [100 MHz, CD_3_OD] δ: 178.07 (C-4), 164.65 (C-7), 161.32 (C-5), 157.62 (C-2), 157.10 (C-9), 148.49 (C-4՛), 144.53 (C-3՛), 134.21 (C-3), 121.79 (C-1՛), 121.63 (C-6`), 116.12 (C-2՛), 114.60 (C-5՛), 104.15 (C-10), 98.51 (C-6), 93.26 (C-8).

Glucose moiety δ: 102.86 (C-1՛՛), 77.01 (C-5՛՛), 76.70 (C-3՛՛), 74.30 (C-2՛՛), 69.78 (C-4՛՛) and 61.11 (C-6՛՛).

# **Discussion about the structure elucidation of the isolated compounds:**

**Compound 1** was obtained as a white amorphous powder (50 mg). It leaves a red spot-on TLC after heating and spraying with 10% H_2_SO_4_ reagent with m.p. (276 °C). The **IR** spectra showed a broad absorption band at 3450.10 cm^-1^ for O-H stretching vibration in addition to absorptions 2924.28, 2854.78 cm^-1^ due to =C-H, C-H stretching vibration of alkanes and at 1636.13 cm^-1^ due to C=C stretching vibrations. The **^1^H-NMR** spectrum of **1** showed the spectrum is featured by eight strong singlet peaks at 1.11 (3H, s), 0.97 (3H, s), 0.82 (3H, s), 0.95 (3H, s), 1.28 (3H, s), 0.85 (3H, s), 1.00 (3H, s), and 0.93 (3H, s), which correspond to methyl groups of C-23 to C-30. Also, the double doublet at δ 5.55 confirms the existence of olefinic proton of C-15, while the multiplet signal at δ 3.23 (1H, m) confirm OH group at C-3. The **^13^C-NMR** spectrum of **1** showed 30 signals with the occurrence of an olefinic carbon resonance between 158.10 and 116.88 suggests a C14-C15 double bond which suggest a taraxerane-type triterpenoid structure, with OH group on C-3 is revealed by signal at δ 79 ppm. Also, the ^13^C-NMR spectrum revealed the presence of eight methyls, ten methylenes, five methines, and eight quaternary carbons for the triterpenoid moiety. **EI-MS** spectrum suggest that chemical formula is triterpene C_30_H_50_O as the molecular ion is *m/z* 426.38 [M]^+^ with peaks 411 [M^+^-CH_3_], 302 [M^+^-C_9_H_16_], 269 [302-(CH_3_+H_2_O)], 204 [M^+^-(C_15_-H_26_O)],189 [204-CH_3_] ^2^. By comparing all spectral data of compound **1** to those described in the literature^3^, compound **1** was identified as Taraxerol.

**Compounds 2 and 3** were positive for Liebermann's and Salkowski's tests, indicating that they are sterols. Compounds 2 and 3 were identified as 3-*β*-stigmast-5-en-3-ol (*β*-sitosterol) and *β-*sitosterol-3-*O*-*β*-D-glucoside (daucosterol), respectively, according to their IR, ^1^HNMR, ^13^CNMR, m.p., and Co-TLC with authentic *β*-sitosterol and *β*-sitosterol-3-*O*-*β*-D-glucoside. This has been verified by comparing their data with those described in the literature^4,5^.

**Compound 4** was obtained as a yellow amorphous powder (55 mg). It leaves a yellow spot-on TLC after spraying with 5% AlCl_3_ reagent with m.p. (318 °C) and **UV** (MeOH) ʎ_max_ at 243, 261 and 300 nm so proposed that compound **4** is flavonoid. The **IR** spectrum showed a broad absorption band at 3416.02 cm^-1^ for O-H stretching vibration in addition to absorptions at 1619.37 cm^-1^ for a carbonyl (C=O) group. **ESI-MS** negative mode mass spectrum of compound **4** displayed peaks at *m/z* 491.3 [M-H^+^]^-^, 537.3 [M+CH_2_O_2_]^-^ proposing C_22_H_20_O_13_ as the chemical formula for compound **4**, which is compatible with Quercetin glucuronide methyl ester. The **^1^H-NMR** spectrum of **4** two doublets and one doublet of doublet signals at δ 7.48, 6.74, 7.49 ppm each integrated for one proton with *J* values = 2.4, 8.8, (2.4, 9.0) Hz for meta and ortho coupling, assigned to H-2՛, H-5՛, and H-6՛, respectively, proving 3՛,4՛ dihydroxylation. Furthermore, 5,7 dihydroxylations confirmed by a meta coupled doublets at δ 6.11, 6.30 ppm (*J* = 2.4 Hz), assigned to H-6 and H-8, respectively, proving 3՛,4՛ dihydroxylation. In addition to a *β*-glycoside linkage is suggested by the large coupling constant of the anomeric proton H-1՛՛ at 5.14 (1H, d, J = 7.8 Hz) with additional signals in the range of 3.20 - 3.48. Whereas **DEPTQ-NMR** spectrum of **4** showed a characteristic carboxylic signal at δ 169.26 supposed glucuronic moiety with ester due to methoxy signal at δ 51.44 alongside **HMBC** spectrum **Figure S4F** confirm 3-*O*-glucuronide-6՛՛ methyl ester by presence of correlation between anomeric proton H-1՛՛ at δ 5.14 with C-3 at δ 134.01 and protons of methoxy group and C-6՛՛ at δ 169.26, respectively. By comparing all of the compound 4's spectral data to those described in the literature^6,7^, compound **4** was identified as quercetin 3-*O*-*β*-D-glucuronide 6՛՛ methyl ester.

**Compound 5** was obtained as a yellow amorphous powder (30 mg). It leaves a yellow spot-on TLC after spraying with 5% AlCl_3_ reagent with m.p. (230 °C). The spectral data of **5** is like those of compound **4** which proposed that **5** is also quercetin derivatives. The **^1^H-NMR** spectrum of **5** supposed a sugar moiety with a *β*-glycoside linkage due to the large coupling constant of the anomeric proton H-1՛՛ at δ 5.15 (1H, d, *J =* 7.52 Hz). Additionally, the C-6՛՛ protons were identified as a pair of two doublets at δ 3.61 (1H, dd, J = 2.4, 11.9 Hz) and 3.47 (1H, dd, J = 5.28, 11.9 Hz). Both had a significant coupling constant of 11.9 Hz, indicating the geminal coupling between two C-6՛՛ proton. Even so, each proton of C-6՛՛ interact separately with the H-5՛՛, with vicinal coupling constants of 2.4 Hz for 6՛՛*α* and 5.28 Hz for 6՛՛*β*. The **APT-NMR** spectrum of **5** showed 21 carbon signals of these, 15 carbon signals were attributed to the aglycone which matching with the reported ^13^C-NMR data of quercetin with downfield chemical shift of C-2 at 157.62 due to hybridization and electronegativity
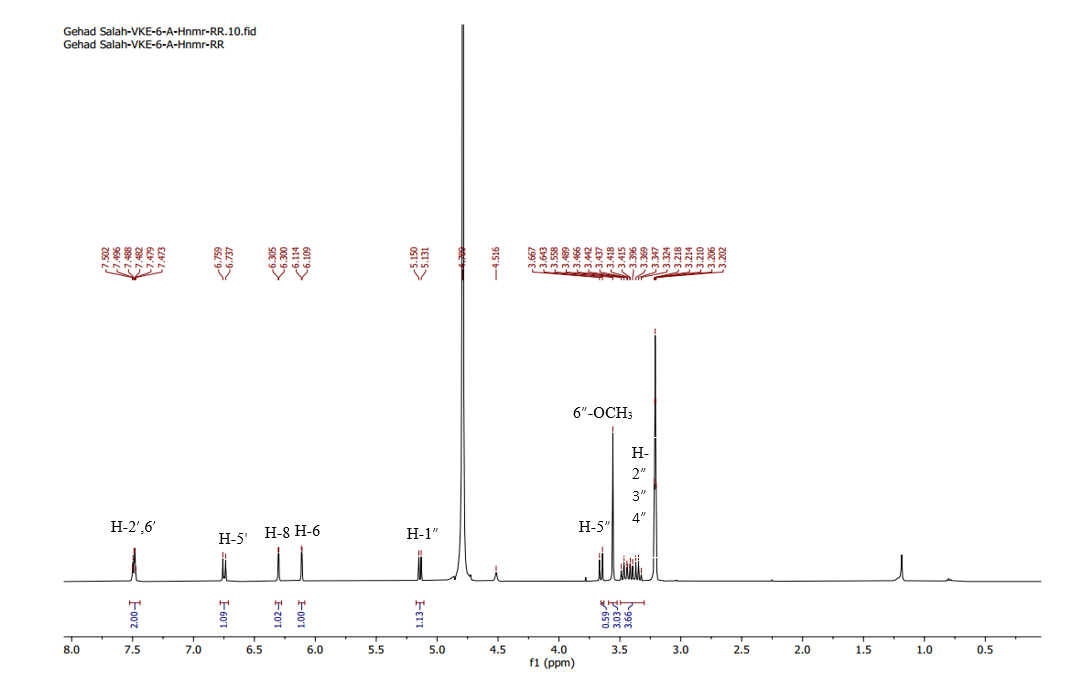
 alongside **HMBC** spectrum **Figure S5F** that reveals two correlations with C-10 at δ 6.30 and 6.11 ppm for H-8 and H-6, respectively, whereas the existence of C-5 is confirmed by a correlation at δ 161.32 ppm with H-6 at δ 6.11 ppm. The remaining 6 carbon signals are due to sugar moiety which confirmed after acid hydrolysis. HMBC spectrum **Figure S5F** confirm the 3-*O*-glucoside by presence of correlation between anomeric proton H-1՛՛ at δ 5.17 and C-3 at 134.21.

ESI-MS negative mode mass spectrum of **5** displayed peaks at m/z 463.3 [M-H^+^]^-^ proposing C_21_H_20_O_12_ as the chemical formula. By comparing all of the compound 5's spectral data to those described in the literature^6,7^, compound **5** was identified as isoquercetin.

**Scheme S1:** Extraction and fractionation steps of *Vitis vinifera* L. var. King Ruby leaves.

**Scheme S2:** Investigation of PE fraction of *Vitis vinifera* L. var. King Ruby leaves.

**Scheme S3:** Column chromatography of USM residue of *Vitis vinifera* L. var. King Ruby leaves.

**Scheme S4:** Column chromatography of MCF residue of *Vitis vinifera* L. var. King Ruby leaves.

**Scheme S5:** Column chromatography of EtOAc residue of *Vitis vinifera* L. var. King Ruby leaves.


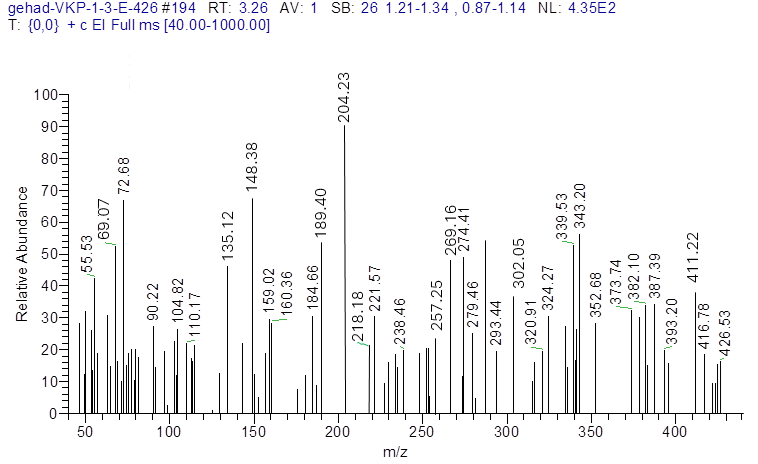

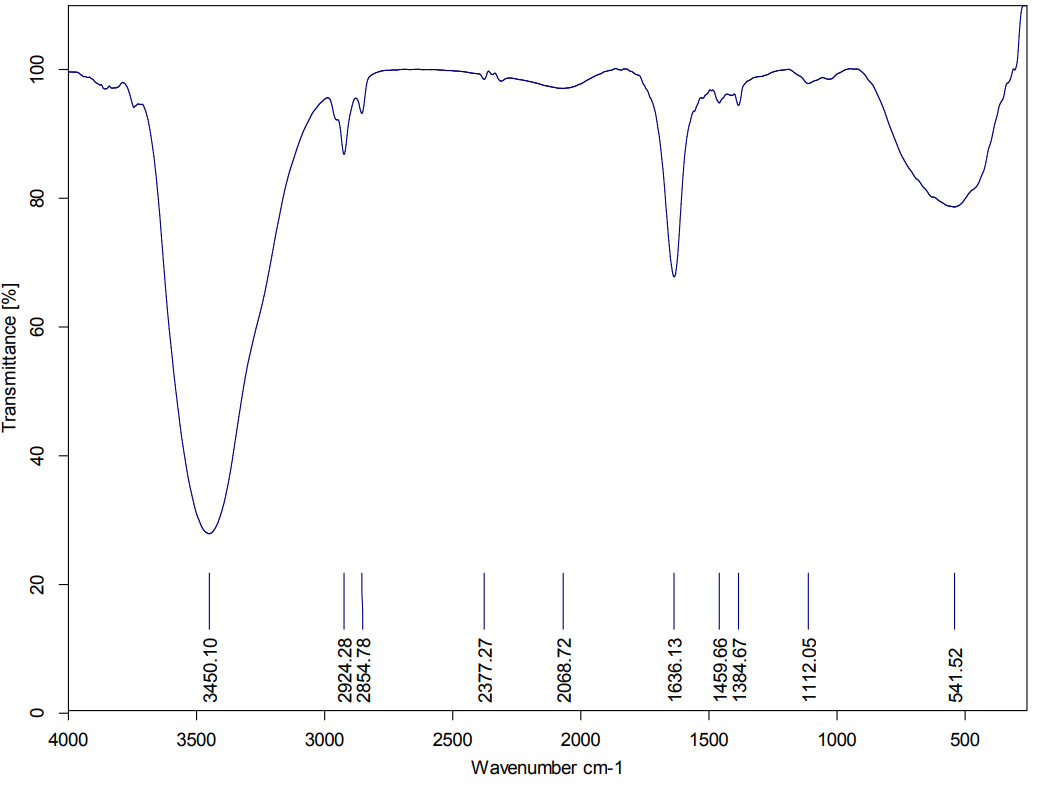


**Figure S1A:** IR spectrum of compound 1 [KBr disc]

**Figure S1B:** EI-MS spectrum of compound **1**


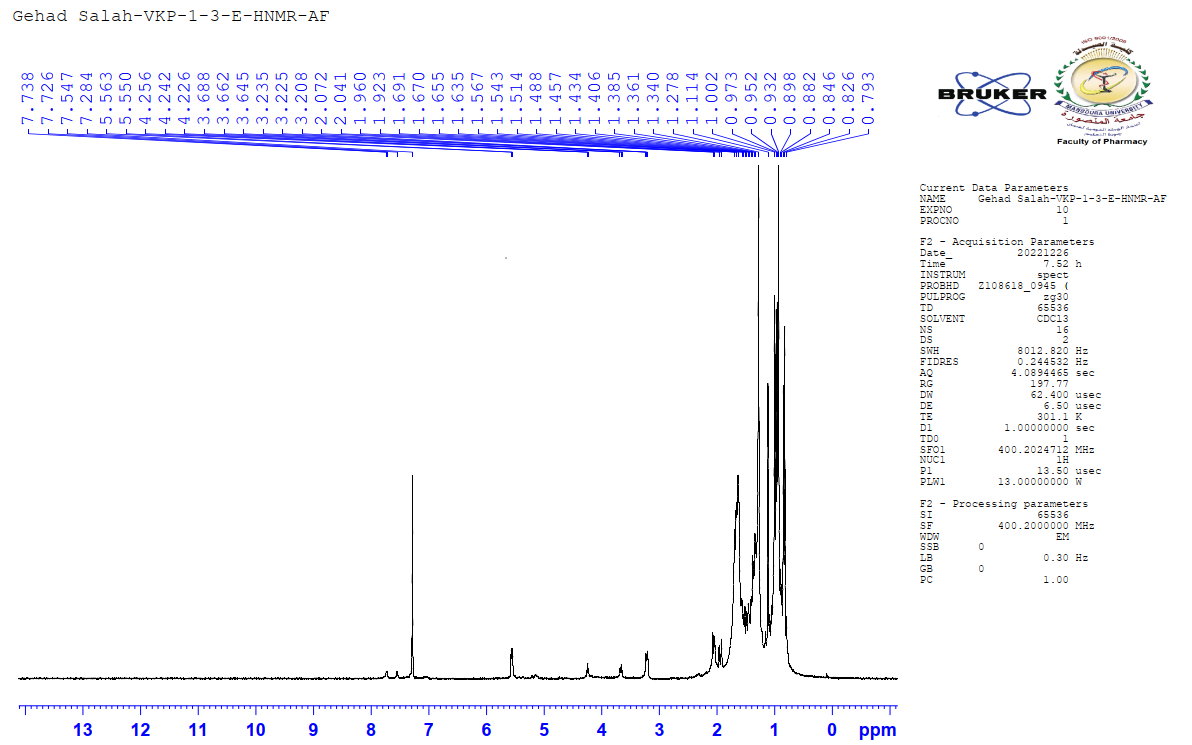

**Figure S1C:** ^1^H-NMR spectrum of compound **1** [CD_3_Cl, 400 MHz]


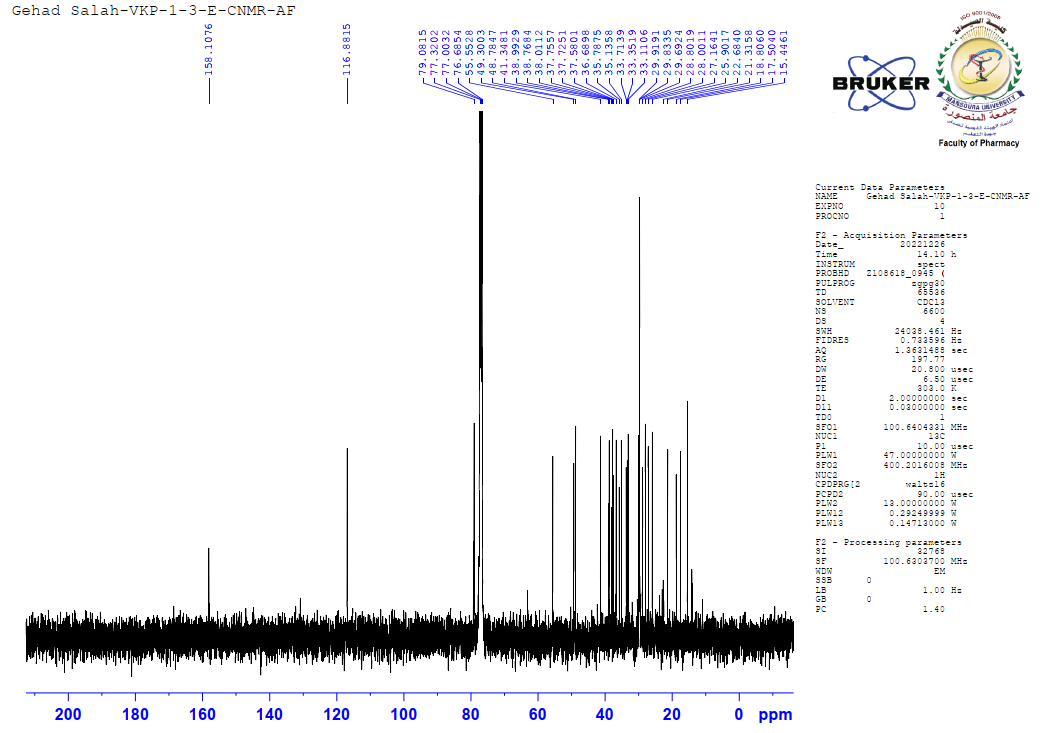

**Figure S1D:** ^13^C-NMR spectrum of compound **1** [CD_3_Cl, 100 MHz]


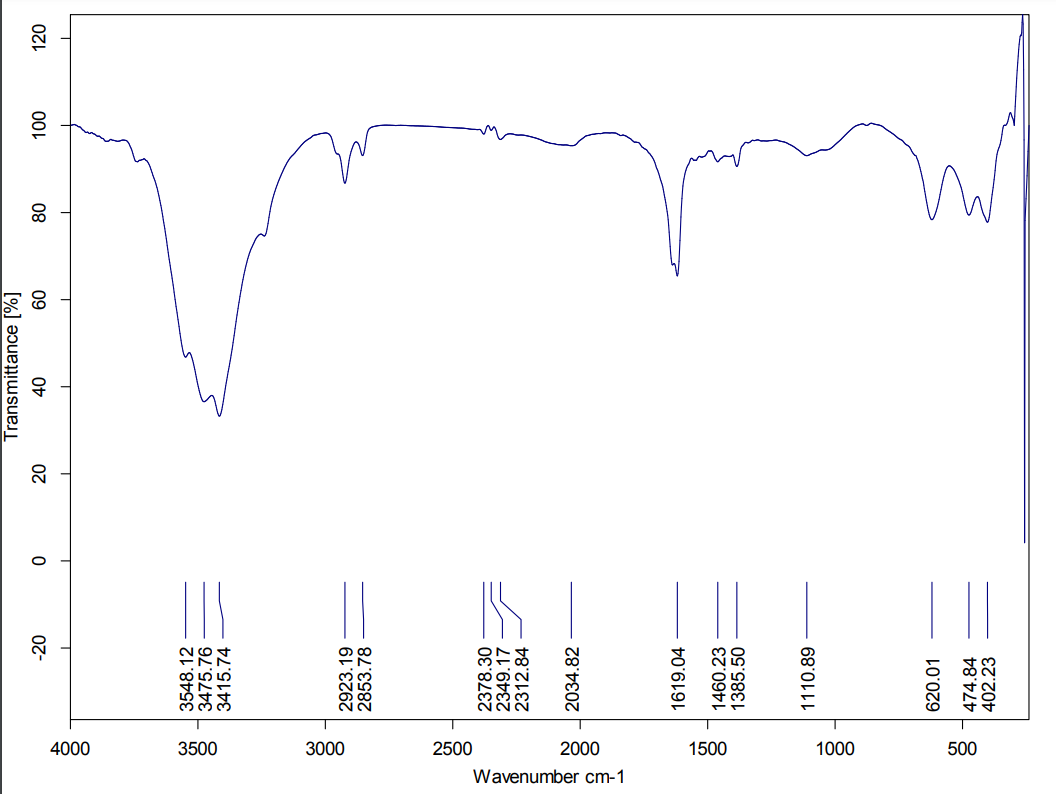


**Figure S2A:** IR spectrum of compound 2 [KBr disc]


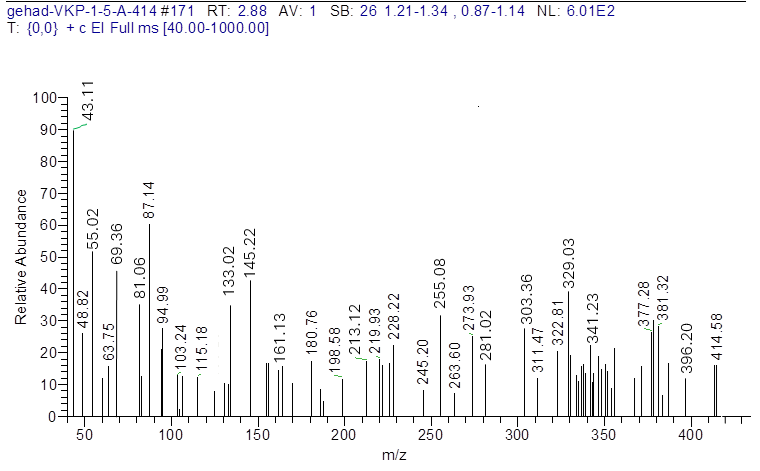


**Figure S2B:** EI-MS spectrum of compound **2**


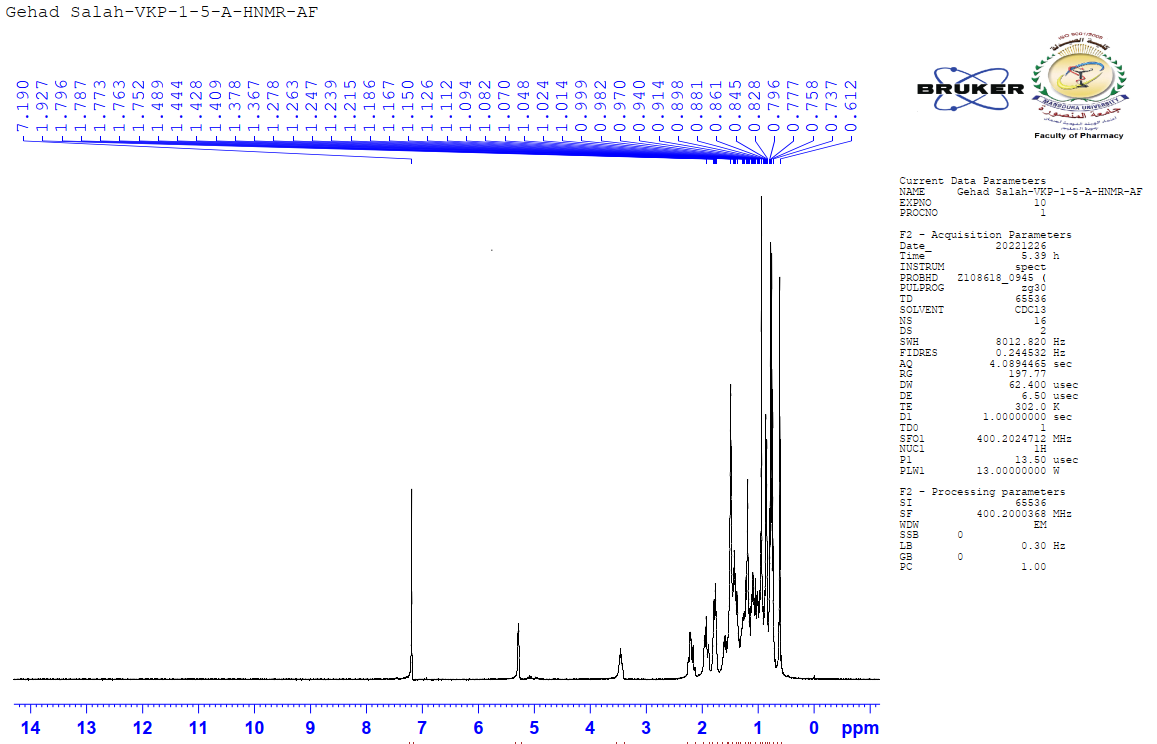

**Figure S2C:** ^1^H-NMR spectrum of compound **2** [CD_3_Cl, 400 MHz]


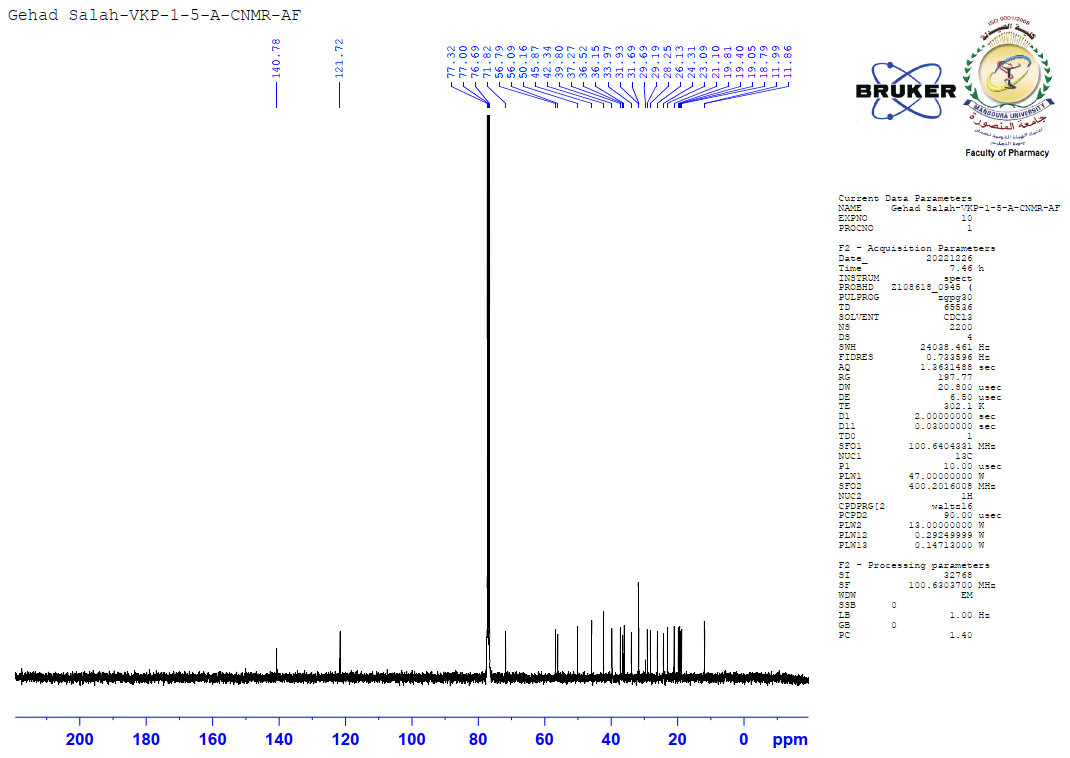

**Figure S2D:** ^13^C-NMR spectrum of compound **2** [CD_3_Cl, 100 MHz]


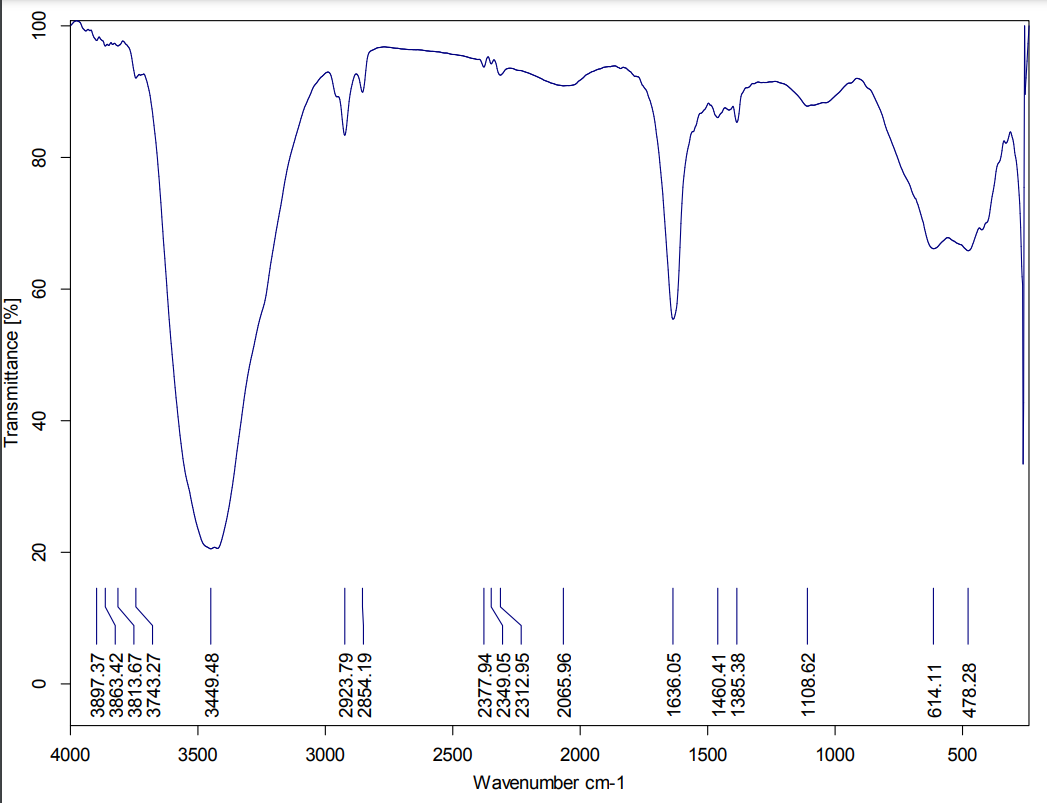


**Figure S3A:** IR spectrum of compound **3** [KBr disc]


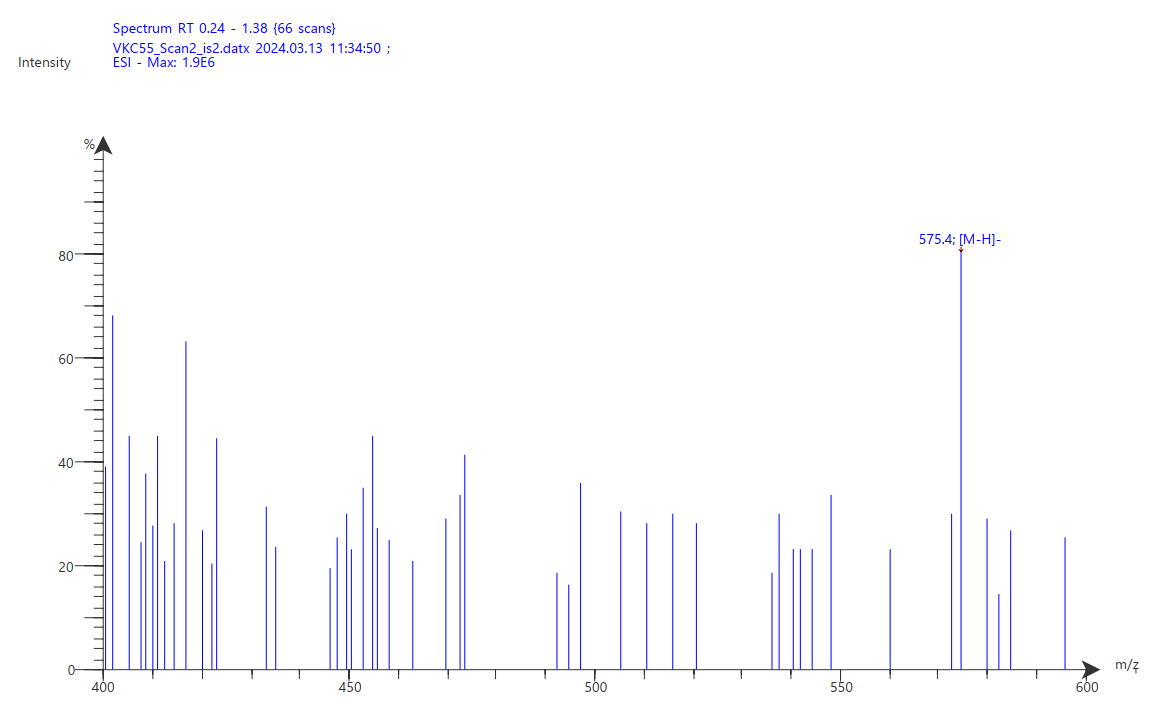


**Figure S3B:** ESI-MS spectrum of compound **3** [Negative mode]


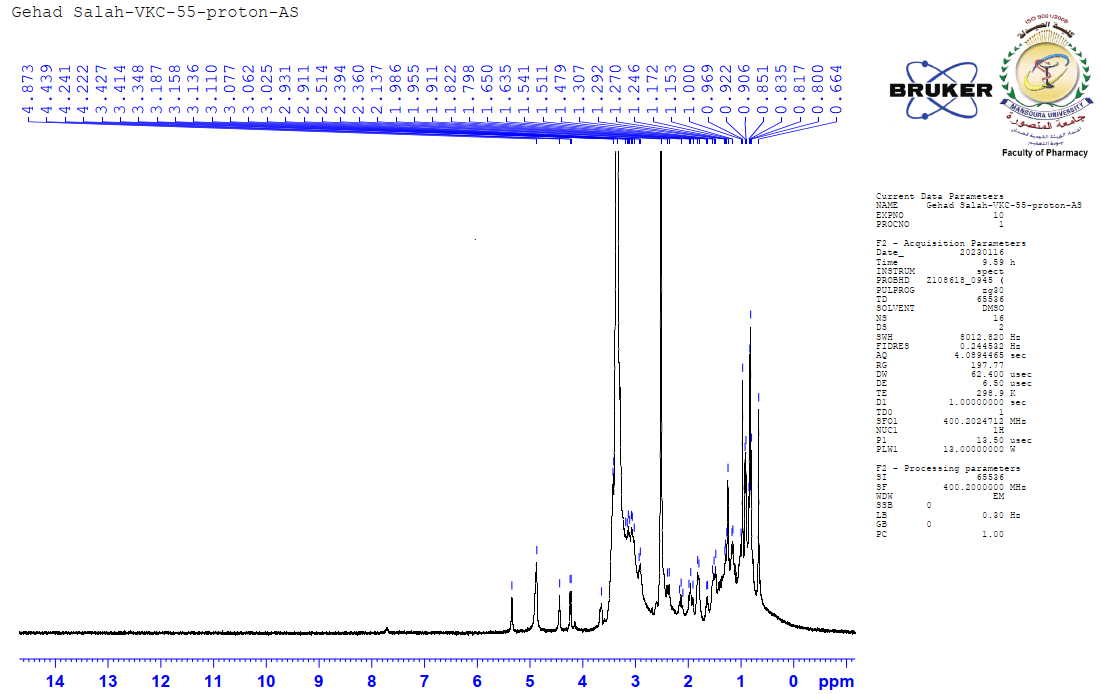

**Figure S3C:** ^1^H-NMR spectrum of compound **3** [DMSO-d_6_, 400 MHz]


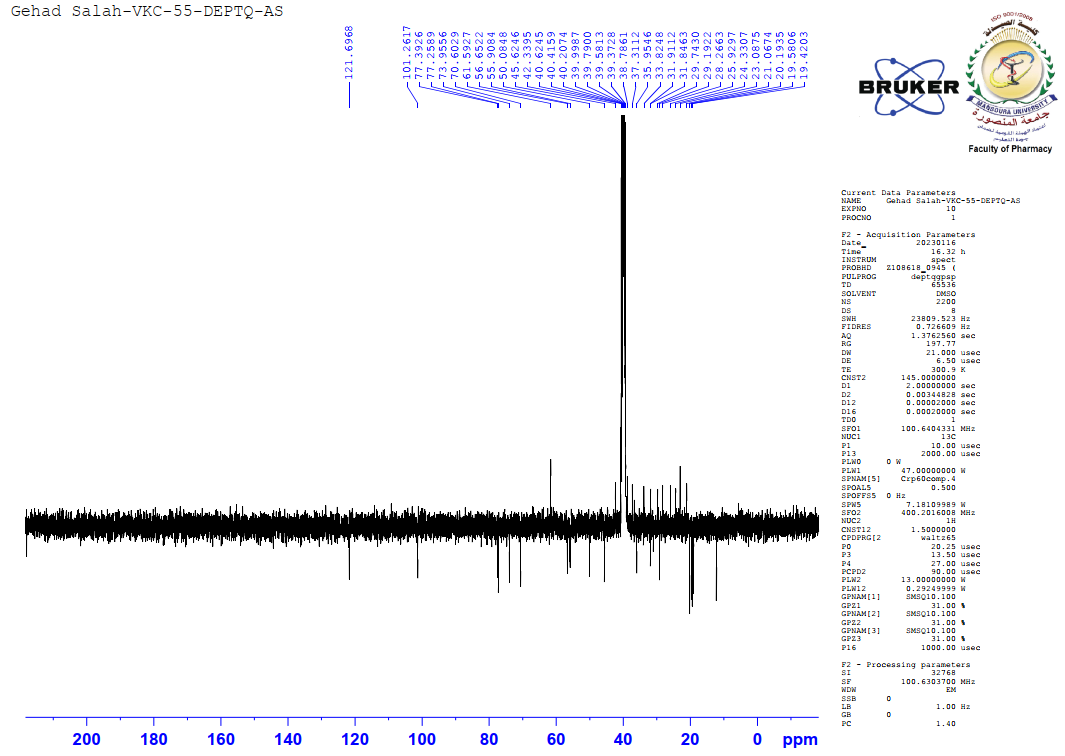

**Figure S3D:** DEPTQ-NMR spectrum of compound **3** [DMSO-d_6_, 100 MHz]

***
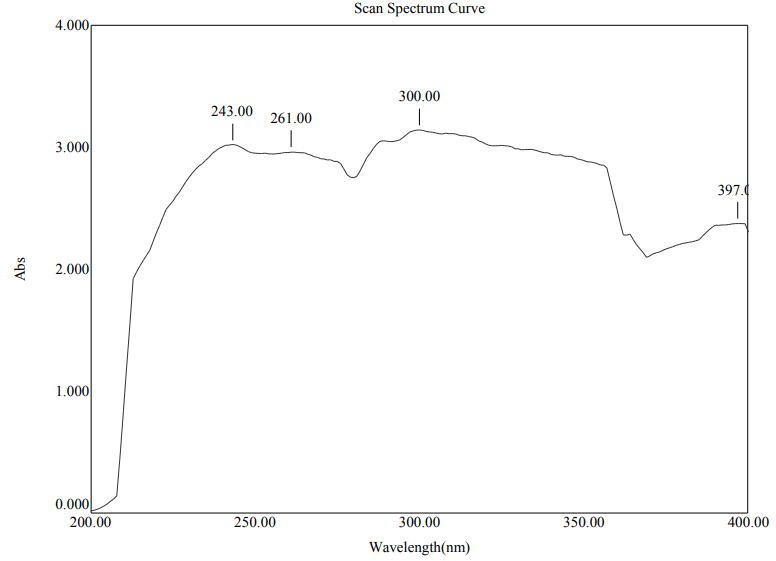
***

**Figure S4A:** UV spectrum of compound **4**


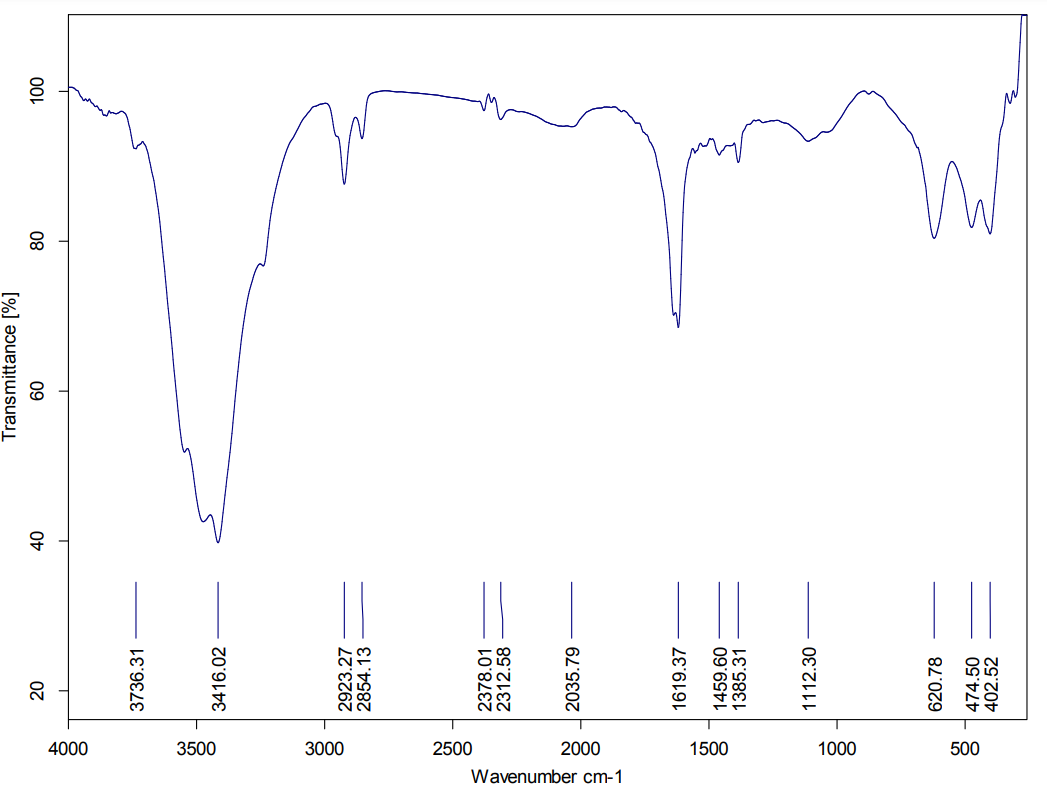


**Figure S4B:** IR spectrum of compound **4** [KBr disc]


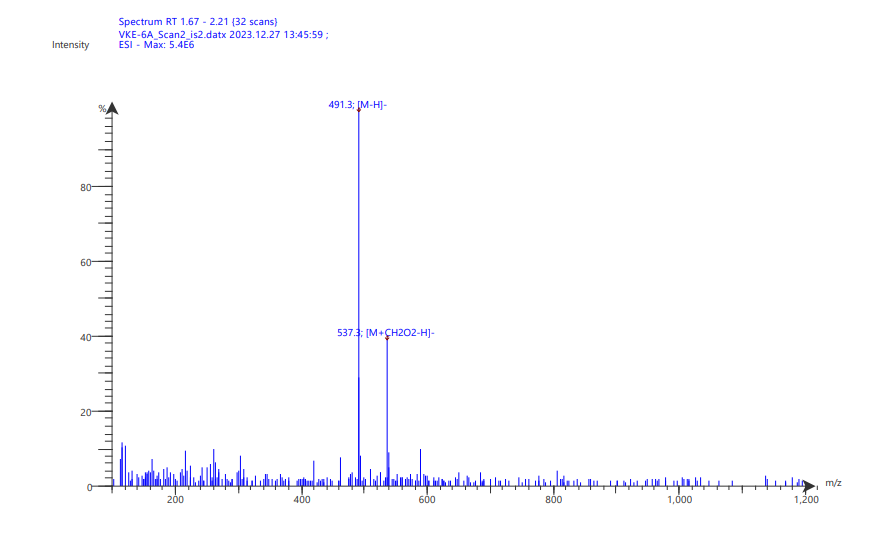


**Figure S4C:** ESI-MS spectrum of compound **4** [Negative mode]


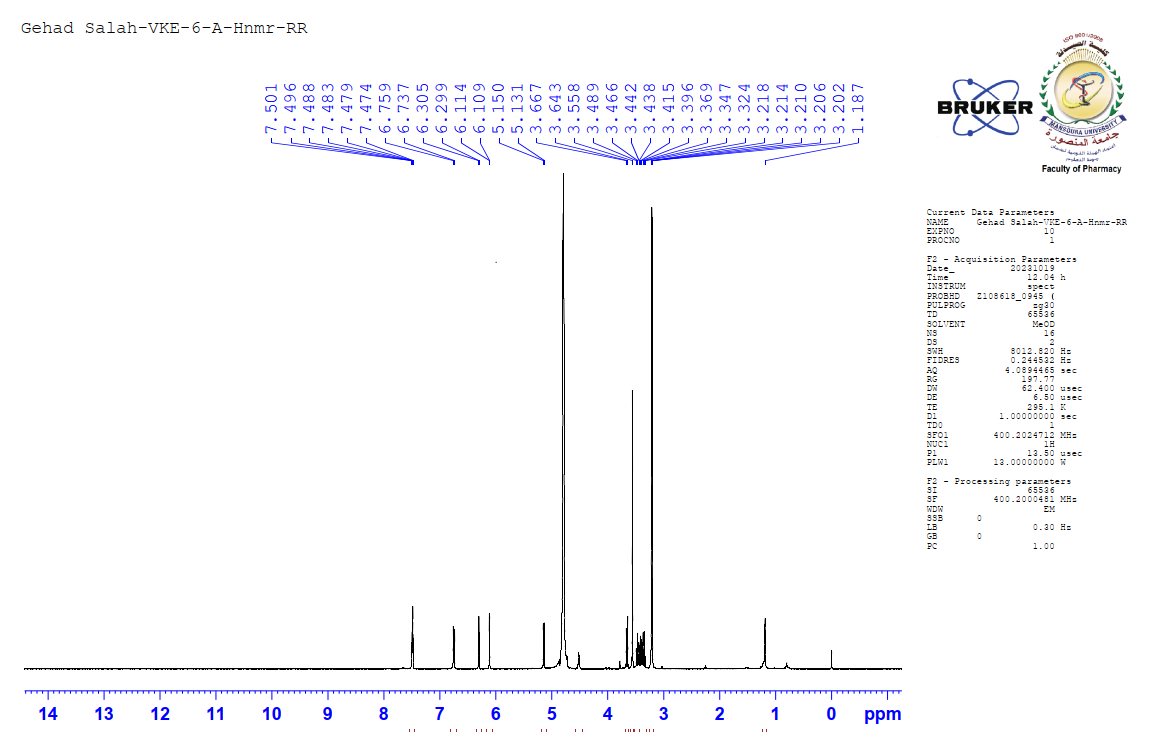

**Figure S4D:** ^1^H-NMR spectrum of compound **4** [CD_3_OD, 400 MHz]


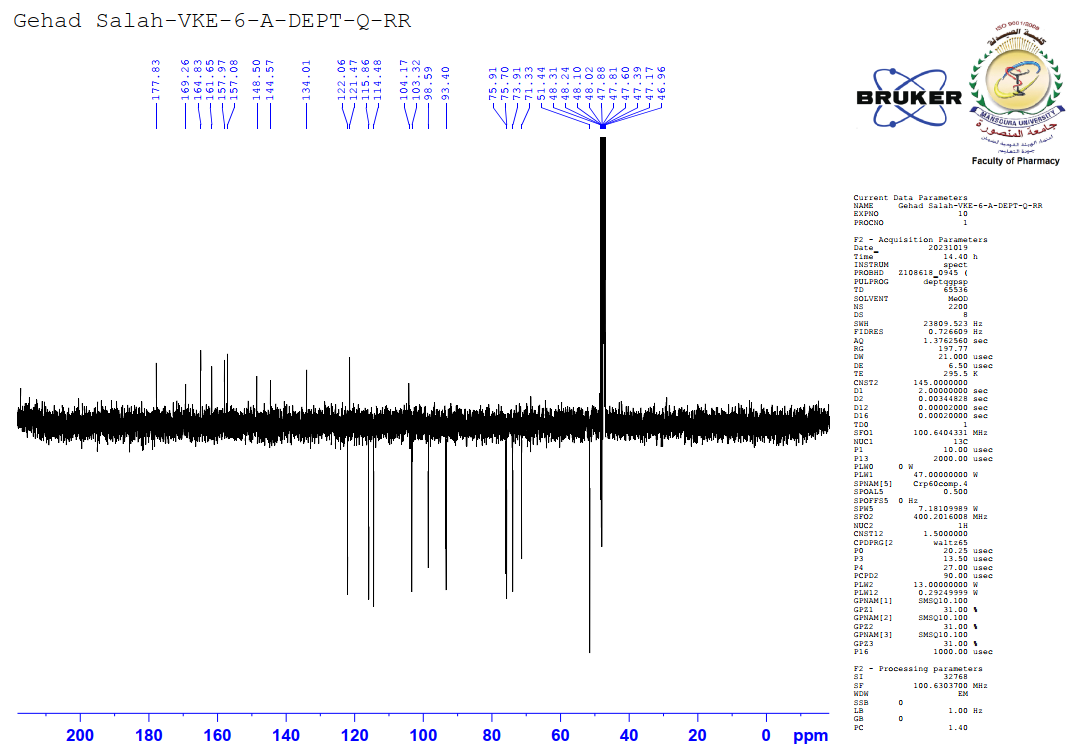


**Figure S4E:** DEPTQ-NMR spectrum of compound **4** [CD_3_OD, 100 MHz]


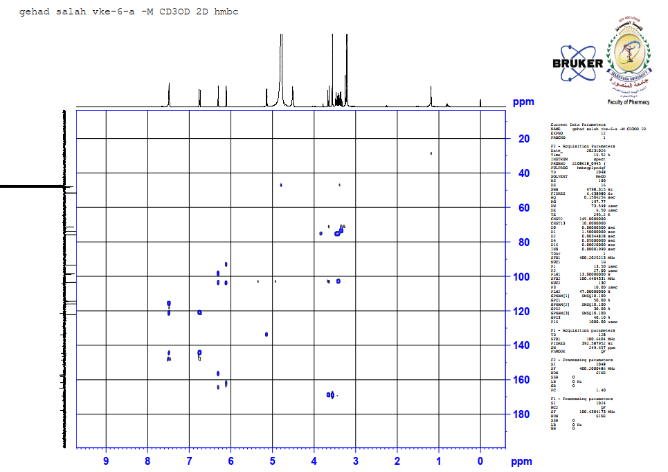


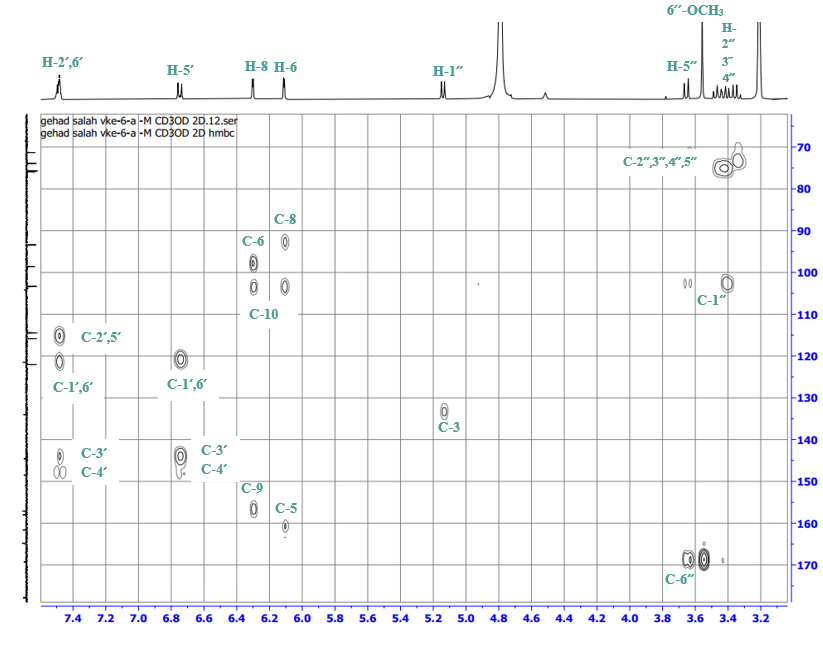


**Figure S4F:** HMBC spectrum of compound **4**

*
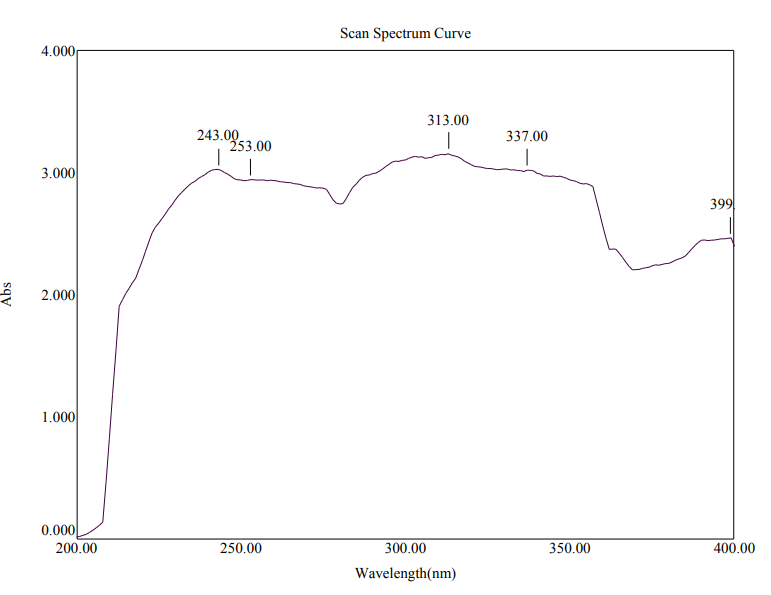
*

**Figure S5A:** UV spectrum of compound **5**

*
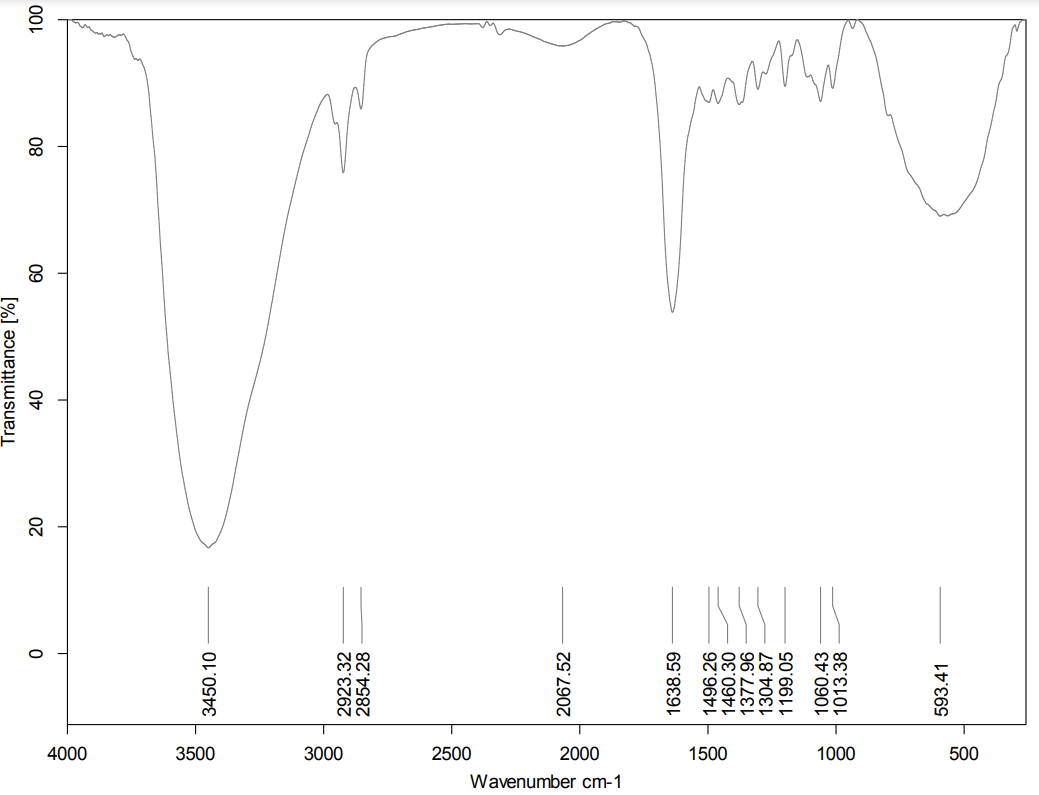
*

**Figure S5B:** IR spectrum of compound **5** [KBr disc]


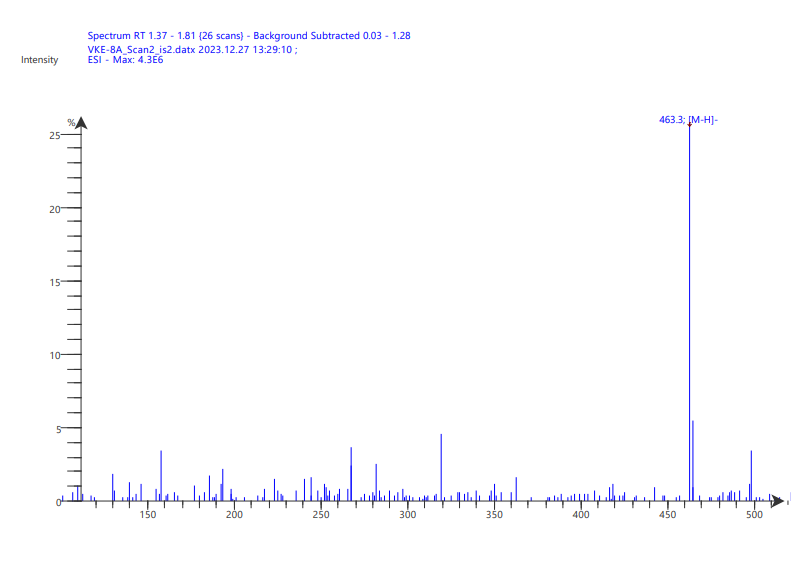


**Figure S5C:** ESI-MS spectrum of compound **5** [Negative mode]

*
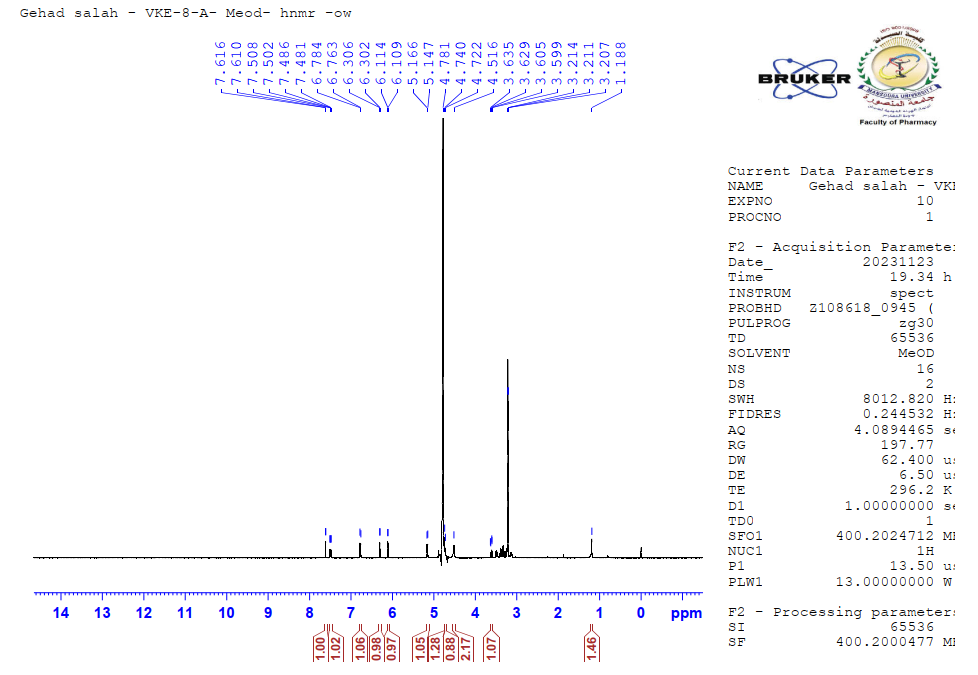
*

**

**Figure S5D:** ^1^H-NMR spectrum of compound **5** [CD_3_OD, 400 MHz]


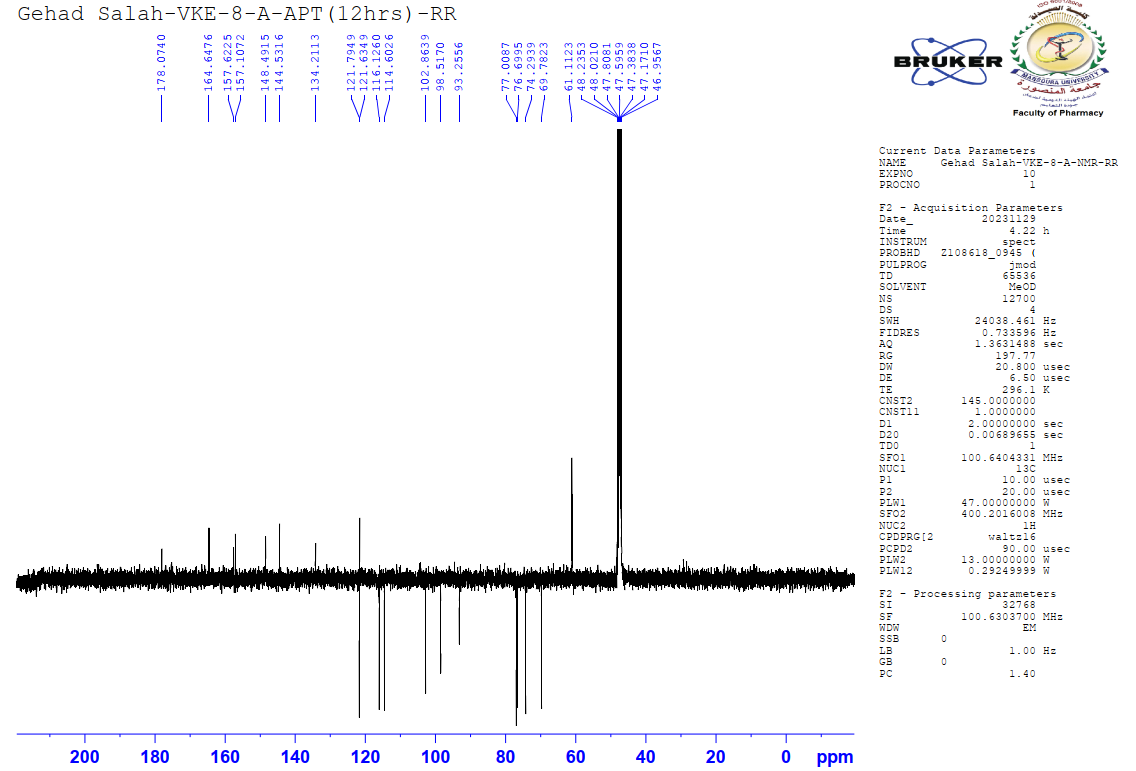

**Figure S5E:** APT-NMR spectrum of compound **5** [CD_3_OD, 400 MHz]


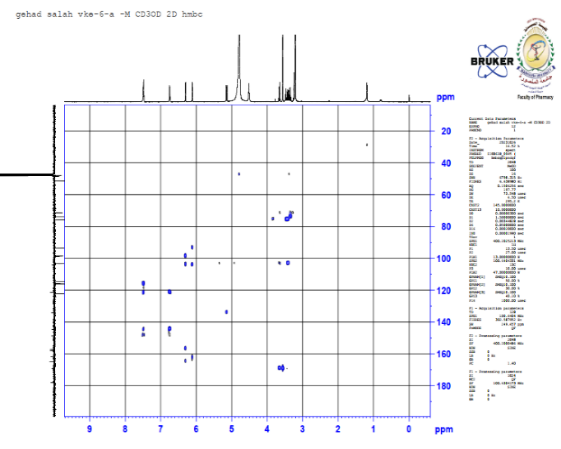


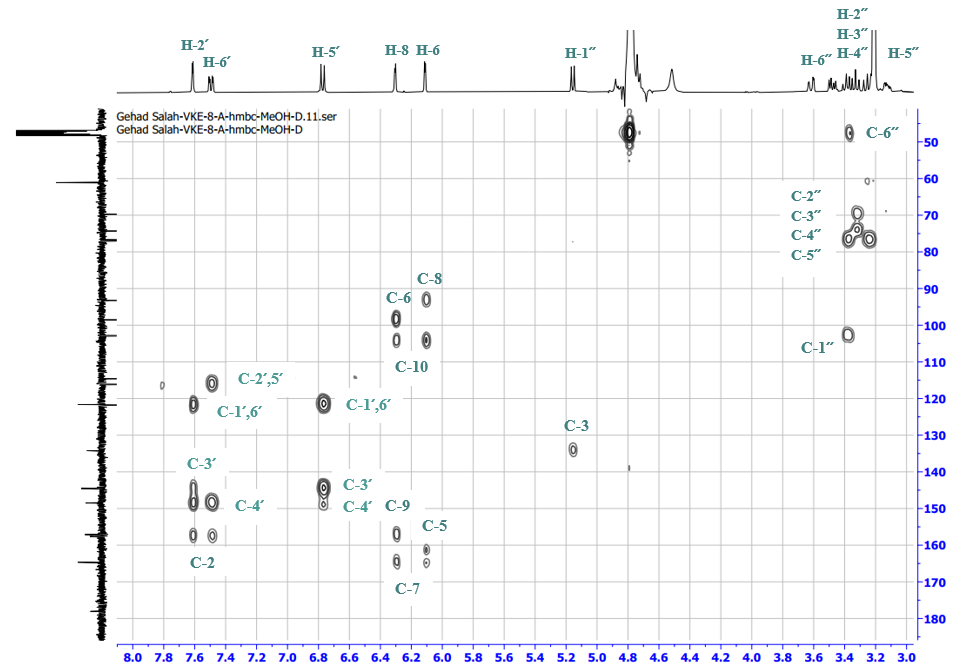


**Figure S5F:** HMBC spectrum of compound **5**


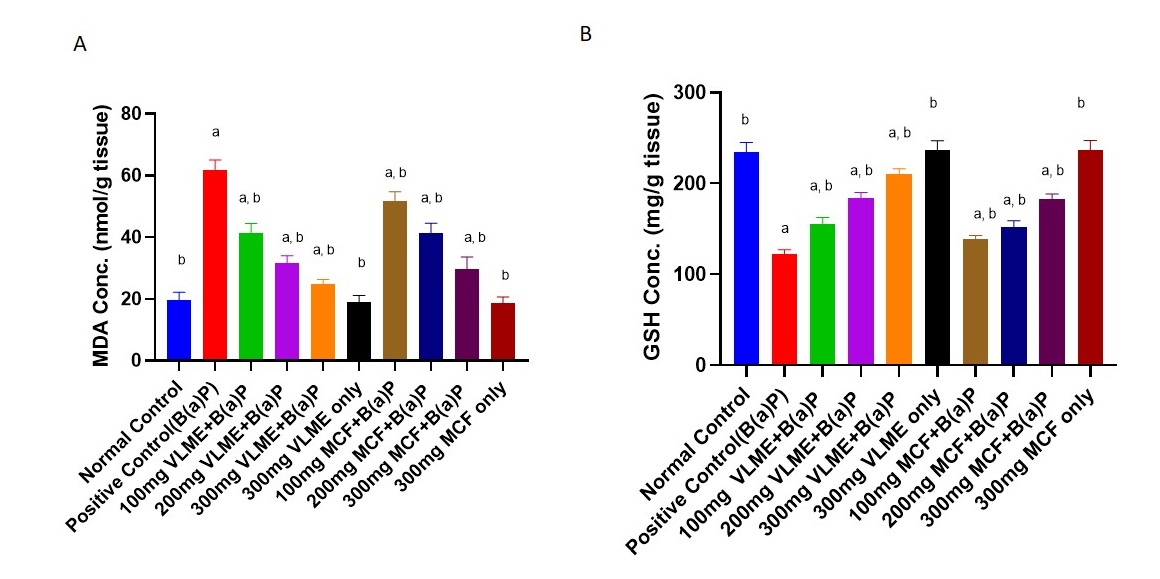


**Figure S1:** Impact of different pre-treatments on **(A)** MDA and **(B)** GSH levels. Data are revealed as mean ± SD, n=6. a: reflects a significant change (p<0.05) against the normal control, b: reflects a significant change (p<0.05) against the positive control group.


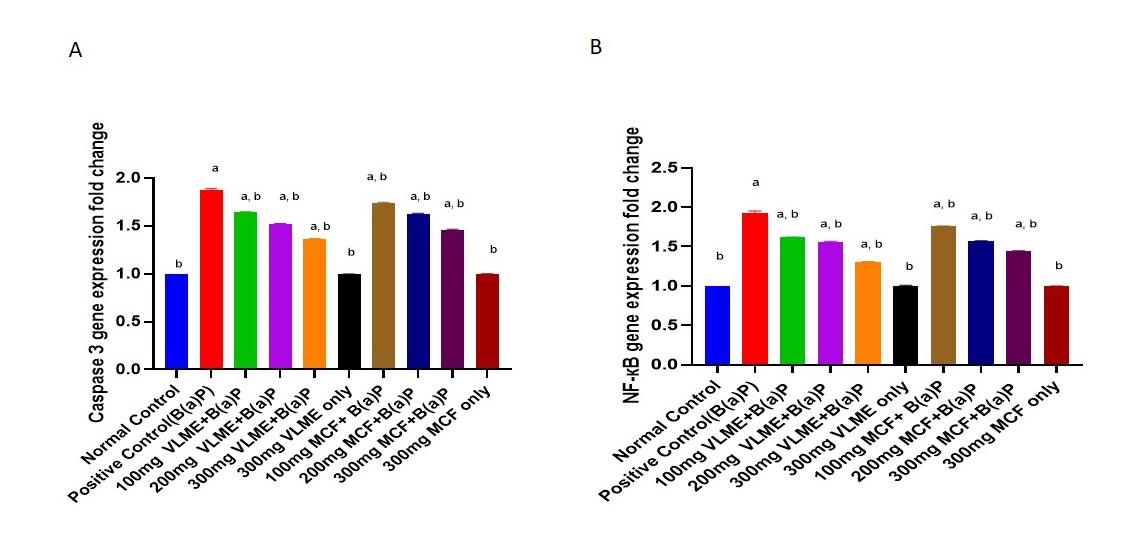


**Figure S2:** Impact of different pre-treatments on **(A)** Caspase 3 and **(B)** NF-ҡB gene expression. Data are revealed as mean±SD, n=6 a: reflects a significant change (p<0.05) against the normal control, b: reflects a significant change (p<0.05) against the positive control group.

# **Table S1: Primer sequences of Caspase 3 and** **NF-кB**

| **Gene** | **Primers** | **References** |
| --- | --- | --- |
| Caspase 3 | GGAGTCTGACTGGAAAGCCGAA  CTTCTGGCAAGCCATCTCCTCA | Casp3 Mouse qPCR Primer Pair  (NM_009810), MP201794, OriGene  Technologies, Inc. |
| NF-кB | AGCGGGAACTGAGTGAGATGA  GCACCCAGGTTGTATCGGG | ^8^ |
| GAPDH | CATCACTGCCACCCAGAAGACTG:  ATGCCAGTGAGCTTCCCGTTCAG | Gapdh Mouse qPCR Primer Pair  (NM_008084), MP205604 OriGene Technologies, Inc. |

# **Supplementary Table SL1:** Phytochemical profiling of VLME by LC-ESI-MS/MS analysis (Negative ESI mode)

| **No** | **R_t_** | **Compound Name** | **m/z** | **Error PPM** | **Formula** | **Ontology** | **MS/MS** | **Ref.** |
| --- | --- | --- | --- | --- | --- | --- | --- | --- |
|  | 1.0329 | D-(+)-Malic acid | 133.0137 | 1.3 | C_4_H_6_O_5_ | Beta hydroxy acids and derivatives | 71.0136  89.02563  115.0035  133.0121 | ^9^ |
|  | 1.0329 | Maleic acid | 115.0032 | 0.5 | C_4_H_4_O_4_ | Dicarboxylic acids and derivatives | 71.01719  115.0081 | [^10^](#_[2]_Attallah_NG,) |
|  | 1.1104 | Myricetin | 317.055 | 0.8 | C_15_H_10_O_8_ | Flavonols | 155.0015  164.9819  273.0826  317.0564 | [^11^](#_[3]_Negm_WA,) |
|  | 1.1104 | D-(-)-Quinic acid | 191.0205 | 0.1 | C_7_H_12_O_6_ | Cycloheanecarboxylic acid (cyclitol) | 85.0273  127.0842  171.0255  173.0472  191.0527 | [^12^](#_[4]_Elmongy_EI,) |
|  | 1.136067 | Trans-ortho-coumaric acid  (2-Hydroxy cinnamic Acid) | 163.0236 | 6.1 | C_9_H_8_O_3_ | Hydroxy cinnamic acids | 76.0931  93.0377  120.0399  163.0249 | ^9^ |
|  | 1.238383 | (+)-3, 3՛, 4՛, 5, 7-Pentahydroxy flavan | 289.0726 | -8.3 | C_15_H_14_O_6_ | Catechins | 205.0321  245.0532  289.0672 | [^13^](#_[5]) |
|  | 1.276883 | Caffeic acid | 179.0562 | 0.3 | C_9_H_8_O_4_ | Hydroxy cinnamic acids | 135.0427  179.0348 | ^10^ |
|  | 1.29005 | (±)-Taxifolin | 303.0827 | 4.8 | C_15_H_12_O_7_ | Flavanonols | 179.0439  190.9552  285.0374  303.0742 | [^14^](#_[6]_Alqahtani_MJ,) |
|  | 1.29005 | (+-)-Jasmonic acid | 209.0665 | 0.9 | C_12_H_18_O_3_ | *α-* Linolenic acid derivatives | 58.0111  165.0219  209.0752 | [^15^](#_[7]_Wilbert_SM,) |
|  | 1.32955 | *γ*-terpinene | 135.0443 | -0.1 | C_10_H_16_ | Branched unsaturated hydrocarbons | 93.0309  121.1238  135.0458 | [^16^](#_[8]_Negm_WA,) |
|  | 1.32955 | Quercetin-3-*O* Arabinoside | 433.0433 | -2.4 | C_20_H_18_O_11_ | Flavonoid-3-*O*-glycosides | 135.0328  271.0003  389.1218  301.0006  433.0428 | ^11^ |
|  | 1.355383 | 3՛-methoxy-4՛, 5, 7-trihydroxyflavonol  (Isorhamnetin) | 315.0316 | 0.4 | C_16_H_12_O_7_ | Flavonols | 246.998  300.0286  315.0316 | ^10^ |
|  | 1.369217 | Quercetin-3-*O*-xyloside | 433.0965 | 0.4 | C_20_H_18_O_11_ | Flavonoid-3-*O*-glycosides | 301.001  364.8968  433.0461 | ^12,^[^17^](#_[10]_El-Shial_EM,) |
|  | 1.38255 | 3-(4-hydroxyphenyl) prop-2-enoic acid  (p-coumaric acid) | 163.038 | 7.8 | C_9_H_8_O_3_ | Hydroxy cinnamic acids | 119.0455  163.043 | [^18^](#_[9]_Attallah_NG,) |
|  | 1.395883 | 2,5-dihydroxybenzoic acid | 153.0176 | 0.5 | C_7_H_6_O_4_ | Hydroxy benzoic acid derivatives | 109.0323  153.0154 | ^14^ |
|  | 1.422883 | Chlorogenic acid | 353.0879 | 3.7 | C_16_H_18_O_9_ | Quinic acids and derivatives | 191.0526  353.0805 | ^19^ |
|  | 1.44955 | Esculin | 339.0726 | 0.5 | C_15_H_16_O_9_ | Coumarin glycosides | 192.2312  270.0071  339.1217 | ^19^ |
|  | 1.751867 | Citraconic acid | 129.0182 | 0 | C_5_H_6_O_4_ | Methyl-branched fatty acids | 129.0436 | ^10^ |
|  | 1.791033 | Hesperetin | 301.0945 | 0.3 | C_16_H_14_O_6_ | 4՛-*O*-methylated flavonoids | 139.0348  273.1385  283.998  301.12045 | ^10^ |
|  | 1.791033 | 3,4-Dihydroxy  phenylacetic acid | 167.0345 | 0.2 | C_8_H_8_O_4_ | Catechols | 123.4149  149.0229  167.02325 | [^20^](#_[12]_Du_J,) |
|  | 2.705983 | P-hydroxybenzoic acid | 137.0244 | -0.8 | C_7_H_6_O_3_ | Hydroxy benzoic acid derivatives | 65.03646  75.02446  93.0338  137.0258 | ^9^ |
|  | 4.42555 | Rosmarinic acid | 359.0789 | -1 | C_18_H_16_O_8_ | Coumaric acids and derivatives | 135.0426  161.0226  179.09  197.039  359.0769 | [^21^](#_[13]_Parejo_I,) |
|  | 4.57905 | 3-Methylxanthine | 165.093 | 0.1 | C_6_H_6_N_4_O_2_ | Xanthines | 95.0579  122.0443  123.0416  165.05625 | [^22^](#_[14]_Liguori_A,) |
|  | 4.668383 | Quercetin-3-glucuronide  (Miquelianin) | 477.0655 | 4.1 | C_21_H_18_O_13_ | Flavonoid-3-*O*-glucuronides | 151.0049  301.0326  477.073 | ^21^ |
|  | 4.681533 | Daphnetin | 177.0176 | 5.8 | C_9_H_6_O_4_ | 7,8-dihydroxy coumarins | 133.0265  149.02926  177.0183 | ^14^ |
|  | 5.059683 | 1-*O*-*β*-D-glucopyranosyl sinapate | 385.1205 | -10.6 | C_17_H_22_O_10_ | Hydroxy cinnamic acid glycosides | 153.0861  190.0221  205.0524  223.0609  385.10698 | ^18^ |
|  | 5.3105 | Kaempferol-3-*O*-glucuronide | 461.0692 | 4.8 | C_21_H_18_O_12_ | Flavonoid-3-*O*-glucuronides | 285.0403  324.9122  461.1833 | ^12^ |
|  | 5.511333 | Quercetin-3,4՛-*O*-di-*β*-glucopyranoside | 625.1432 | -0.7 | C_27_H_30_O_17_ | Flavonoid-3-*O*-glycosides | 301.0349  625.1389 | ^18^ |
|  | 5.764483 | Okanin-4՛-*O*-glucoside  (Marein) | 449.1088 | 0.7 | C_21_H_22_O_11_ | Flavonoid-*O*-glycosides | 151.0051  285.0583  303.0601  403.1754  431.1761  449.0966 | ^18^ |
|  | 5.85315 | Delphinidin-3-*O*-(6՛՛-*O*-*α*-rhamnopyranosyl-*β*-glucopyranoside)  (Tulipanin) | 609.146 | -0.4 | C_27_H_31_O_16_ | Anthocyanidin-3-*O*-glycosides | 302.415  609.1414 | [^23^](#_[15]_Hegazy_MM,) |
|  | 5.865817 | Delphinidin | 302.1664 | 4.1 | C_15_H_11_O_7_ | Anthocyanidin | 153.0118  173.0317  229.0567  257.0092  302.1664 | [^24^](#_[16]_Scorrano_S,) |
|  | 6.0838 | Quercetin-3-*O*-galactoside  (Hyperoside) | 463.0884 | -0.2 | C_21_H_20_O_12_ | Flavonoid-3-*O*-glycosides | 151.0034  255.0256  271.0238  301.0168  463.0839 | ^21^ |
|  | 6.58495 | Isorhamnetin-3-*O*-rutinoside  (Narcissin) | 623.1636 | -0.4 | C_28_H_32_O_16_ | Flavonoid-3-*O*-glycosides | 315. 0575  577.2865  623.1983 | ^10,12^ |
|  | 6.60995 | 3-(4-hydroxy-3,5-dimethoxyphenyl)-2-propenoic acid | 223.1336 | 0.9 | C_11_H_12_O_5_ | Hydroxy cinnamic acids | 191.1036  223.1323 | ^10^ |
|  | 6.60995 | Kaempferol-3-*O*-(6-p-coumaroyl)-glucoside | 593.117 | 0.1 | C_30_H_26_O_13_ | Flavonoid-3-*O*-p-coumaroyl glycosides | 241.0438  285.0401  593.1488 | ^10^ |
|  | 6.718433 | Kaempferol-3-*O*-glucoside  (Astragalin) | 447.0944 | -0.7 | C_21_H_20_O_11_ | Flavonoid-3-*O*-glycosides | 227.0331  255.03  285.0261  447.0935 | ^21^ |
|  | 6.842267 | Isorhamnetin-3-*O*-glucoside | 477.1019 | 2.8 | C_22_H_22_O_12_ | Flavonoid-3-*O*-glycosides | 315.0481  477.1009 | ^12^ |
|  | 6.992084 | P-Nitrophenol | 138.0199 | -0.8 | C_6_H_5_NO_3_ | Nitrophenols | 92.02218  108.0212  138.0182 | [^25^](#_[17]_Mahrous_FS,) |
|  | 7.129583 | Quercetin-3-*O*-glucoside  (Isoquercitrin) | 463.2183 | 0.9 | C_21_H_20_O_12_ | Flavonoid-3-*O*-glycosides | 255.0302  271.0309  301.0427  300.0378  463.0809 | ^21^ |
|  | 7.179584 | Rhoifolin | 577.1517 | -0.3 | C_27_H_30_O_14_ | Flavonoid-7-*O*-glycosides | 508.8501  577.1487 | [^26^](#_[18]_Alotaibi_B,) |
|  | 7.358233 | E-3, 4, 5՛-Trihydroxy-3՛-glucopyranosyl stilbene  (E-Astringin) | 405.1684 | -0.3 | C_20_H_22_O_9_ | Stilbene glycosides | 190.9516  243.0675  405.1926 | ^12^ |
|  | 7.3964 | Daidzein-8-*C*-glucoside  (Puerarin) | 415.1958 | 3.9 | C_21_H_20_O_9_ | Isoflavonoid-*C*-glycosides | 179.0516  253.0801  369.1492  415.1465 | [^17^](#_[10]_El-Shial_EM,) |
|  | 7.4344 | Phlorizin | 435.1287 | 1.1 | C_21_H_24_O_10_ | Flavonoid-*O*-glycosides | 125.0233  389.2193  435.1286 | ^11^ |
|  | 7.523567 | Kaempferol-3-*O*-*α*-L-rhamnoside | 431.0999 | -1.1 | C_21_H_20_O_10_ | Flavonoid-3-*O*-glycosides | 341.0401  362.8923  385.2207  431.1906 | ^10^ |
|  | 7.86155 | 4-deoxyphloridzin | 419.2253 | 0.4 | C_21_H_24_O_9_ | Flavonoid *O*-glycosides | 257.0863  351.1492  419.2194 | ^16^ |
|  | 8.576 | 3-(4-hydroxy-3-methoxyphenyl) prop-2-enoic acid | 193.0503 | 1 | C_10_H_10_O_4_ | Hydroxy cinnamic acids | 161.103  193.0514 | [^17^](#_[10]_El-Shial_EM,) |
|  | 9.53695 | Naringenin | 271.0587 | 8.5 | C_15_H_12_O_5_ | Flavanones | 151.008  271.0653 | ^21^ |
|  | 10.03327 | Resveratrol | 227.0709 | 1.1 | C_14_H_12_O_3_ | Stilbenes | 158.9789  185.0573  227.0789 | [^27^](#_[19]_Kadam_D,) |
|  | 10.05827 | Apigenin | 269.0443 | 1.4 | C_15_H_10_O_5_ | Flavones | 269.0488 | ^12^ |
|  | 10.53973 | Luteolin | 285.0407 | -0.2 | C_15_H_10_O_6_ | Flavones | 269.158  285.0401 | ^12^ |
|  | 12.56163 | Quercetin | 301.1119 | -0.2 | C_15_H_10_O_7_ | Flavonols | 255.2372  301.0005 | ^12^ |
|  | 18.00268 | *γ*-Linolenic acid | 277.2179 | -0.9 | C_18_H_30_O_2_ | Lineolic acids and derivatives | 277.2149 | ^10^ |

# **Supplementary Table** **SL2:** Phytochemical profiling of VLME by LC-ESI-MS/MS analysis (Positive ESI mode)

| **No** | **R_t_** | **Title** | **m/z** | **Error PPM** | **Formula** | **Ontology** | **MSMS** | **Ref.** |
| --- | --- | --- | --- | --- | --- | --- | --- | --- |
|  | 1.042283 | 4-Aminophenol | 110.0095 | -6.4 | C_6_H_7_NO | Substituted anilines | 66.02007  68.98297  80.20762  110.00999 | ^28^ |
|  | 1.170117 | Choline | 104.1061 | 2.2 | C_5_H_14_NO | Cholines | 58.06492  60.08139  104.10792 | ^9^ |
|  | 1.283767 | Trigonelline | 138.0526 | 6.9 | C_7_H_7_NO_2_ | Alkaloids | 92.05025  94.06629  120.0457  138.05241 | ^29^ |
|  | 1.2966 | (+)-3, 3՛, 4՛, 5, 7-Pentahydroxyflavan | 291.104 | 0.8 | C_15_H_14_O_6_ | Catechins | 291.1032 | ^11^ |
|  | 1.424267 | Resveratrol | 229.1538 | -2 | C_14_H_12_O_3_ | Stilbenes | 58.06255  70.06614  229.15623 | ^11^ |
|  | 1.681917 | 3՛, 4՛, 5, 7-tetrahydroxy flavanone | 289.0718 | -0.2 | C_15_H_12_O_6_ | Flavanones | 253.0980  271.1659  289.06387 | ^11^ |
|  | 4.6076 | Caffeine | 195.0878 | -0.8 | C_8_H_10_N_4_O_2_ | Alkaloids | 138.0623  163.0392  195.08462 | ^11^ |
|  | 4.971917 | Genistein | 271.1176 | -0.3 | C_15_H_10_O_5_ | Isoflavones | 91.05318  119.0883  271.08214 | ^29^ |
|  | 5.115233 | 6,7-dihydroxy coumarin  (Esculetin) | 179.0322 | 3.1 | C_9_H_6_O_4_ | 6,7-dihydroxy coumarins | 51.02271  77.03749  179.03142 | ^11^ |
|  | 5.2849 | Nicotinamide | 123.0799 | 0.1 | C_6_H_6_N_2_O | Nicotinamides | 78.03477  80.04845  123.08806 | ^29^ |
|  | 5.921834 | Procyanidin B2 | 579.1841 | -0.7 | C_30_H_26_O_12_ | Biflavonoids and polyflavonoids | 112.0905  579.17452 | ^11^ |
|  | 5.9467 | Apigenin 8-*C*-glucoside (Vitexin) | 433.1486 | -1 | C_21_H_20_O_10_ | Flavonoid-8-*C*-glycosides | 271.0587  415.1675  433.13925 | ^29^ |
|  | 5.9762 | Gossypin | 481.1043 | -10.5 | C_21_H_20_O_13_ | Flavonoid-8-*O*-glycosides | 319.0453  481.17694 | ^26^ |
|  | 6.026516 | Myricetin | 319.0443 | -0.4 | C_15_H_10_O_8_ | Flavonols | 153.01113  245.04859  319.05561 | ^26^ |
|  | 6.415667 | Delphinidin-3-*O*-(6՛՛-*O*-*α*-rhamnopyranosyl-*β*-glucopyranoside)  (Tulipanin) | 611.1587 | 2.9 | C_27_H_31_O_16_ | Anthocyanidin-3-*O*-glycosides | 303.04822  465.0953  611.18283 | [^30^](#_[22]_Bochi_VC,) |
|  | 6.415667 | Diosmin | 609.1943 | 0.6 | C_28_H_32_O_15_ | Flavonoid-7-*O*-glycosides | 286.0991  609.18745 | ^9^ |
|  | 6.558 | Trans-cinnamate | 149.0946 | 0.7 | C_9_H_8_O_2_ | Cinnamic acids | 93.0311  121.02248  149.02013 | ^25^ |
|  | 6.723817 | Daidzein-8-*C*-glucoside  (Puerarin) | 417.1556 | -2.1 | C_21_H_20_O_9_ | Isoflavonoid-*C*-glycosides | 180.9983  399.25169  417.15025 | ^11^ |
|  | 6.780983 | Quercetin-3-*O*-galactoside  (Hyperoside) | 465.0999 | 4.5 | C_21_H_20_O_12_ | Flavonoid-3-*O*-glycosides | 165.02172  303.05362  465.15691 | ^26^ |
|  | 6.805984 | (±)-Taxifolin | 305.0564 | -0.2 | C_15_H_12_O_7_ | Flavanonols | 305.05263 | ^11^ |
|  | 6.918817 | Quercetin-3-*O*-arabinoside | 435.1619 | 0.4 | C_20_H_18_O_11_ | Flavonoid-3-*O*-glycosides | 220.17426  303.00446  435.14691 | [^31^](#_[23]_Santos_MC,) |
|  | 6.995467 | Maritimetin-6-*O*-glucoside | 449.1765 | 0 | C_21_H_20_O_11_ | Aurone-*O*-glycosides | 241.0466  269.1746  287.0509  449.1791 | ^26^ |
|  | 7.075967 | Isosakuranetin-7-*O*-neohesperidoside  (Poncirin) | 595.1608 | 6.4 | C_28_H_34_O_14_ | Flavonoid-7-*O*-glycosides | 129.0552  153.0565  287.0554  433.1160  449.1128  595.1606 | ^9^ |
|  | 7.154133 | Isorhamnetin-3-*O*-rutinoside | 625.1744 | -0.1 | C_28_H_32_O_16_ | Flavonoid-3-*O*-glycosides | 129.05552  317.06642  479.12322  625.17771 | ^29^ |
|  | 7.180467 | Cyanidin-3-*O*-glucoside | 449.1079 | 0 | C_21_H_21_O_11_ | Anthocyanidin-3-*O*-glycosides | 85.02554  147.05803  287.05557  449.10706 | ^30^ |
|  | 7.396783 | Luteolin | 287.0558 | -0.4 | C_15_H_10_O_6_ | Flavones | 147.10597  153.01831  287.0606 | ^26^ |
|  | 7.396783 | Kaempferol-3-*O*-glucoside (Astragalin) | 449.1102 | -3.7 | C_21_H_20_O_11_ | Flavonoid-3-*O*-glycosides | 287.05517  449.11239 | [^32^](#_[24]_Chen_S,) |
|  | 7.525283 | 3՛-methoxy-4՛, 5, 7-trihydroxyflavono (Isorhamnetin) | 317.0628 | 2.6 | C_16_H_12_O_7_ | Flavonols | 165.06808  271.16138  317.06631 | ^26^ |
|  | 7.538116 | Quercetin-3-*O*-glucuronide | 479.0815 | 0.6 | C_21_H_18_O_13_ | Flavonoid-3-*O*-glucuronides | 135.05247  303.04794  479.08522 | ^31^ ^11^ |
|  | 7.588367 | Naringenin-7-*O*-glucoside | 435.1341 | -4.6 | C_21_H_22_O10 | Flavonoid-7-*O*-glycosides | 147.04075  153.01806  273.07597  435.10495 | ^26^ |
|  | 7.949083 | Okanin-4՛-*O*-glucoside | 451.1719 | 0 | C_21_H_22_O_11_ | Flavonoid *O*-glycosides | 289.15512  451.18008 | [^33^](#_[25]_Gouda_HM,) |
|  | 7.961417 | 3-Formylindole | 146.0618 | -3.9 | C_9_H_7_NO | Indoles | 118.0582  146.05965 | ^33^  [^34^](#_[26]_Upreti_M,) |
|  | 8.151584 | Kaempferol-3-*O*-glucuronide | 463.0854 | 0.8 | C_21_H_18_O_12_ | Flavonoid-3-*O*-glucuronides | 287.05542  463.08746 | [^32^](#_[24]_Chen_S,) |
|  | 9.1717 | Ononin | 431.1439 | 2.4 | C_22_H_22_O_9_ | Isoflavonoid-*O*-glycosides | 268.10985  431.1377 | [^35^](#_[27]_Shen_J,) |
|  | 9.444517 | Quercetin | 303.0984 | 0.1 | C_15_H_10_O_7_ | Flavonols | 153.0181  181.1541  303.0483 | ^26^ |
|  | 10.65728 | Acacetin | 285.1477 | -0.4 | C_16_H_12_O_5_ | 4՛-*O*-methylated flavonoids | 153.0565  187.1091  242.0999  270.0369  285.13988 | ^9^ |
|  | 11.0791 | Methyl jasmonate | 225.11 | -0.7 | C_13_H_20_O_3_ | Jasmonic acids | 151.0265  225.10822 | ^15^ |
|  | 11.1613 | Linoleic acid | 281.1365 | -0.6 | C_18_H_32_O_2_ | Lineolic acids and derivatives | 112.88699  151.0366  281.13365 | ^11^ |
|  | 11.41423 | Chlorogenic acid | 355.1524 | -0.8 | C_16_H_18_O_9_ | Quinic acids and derivatives | 147.04426  355.14821 | ^26^ |
|  | 13.44897 | Nicotine | 163.1103 | 14.8 | C_10_H_14_N_2_ | Alkaloids | 130.06813  132.63487  163.04303 | [^36^](#_[28]_Smyth_TJ,) |
|  | 13.96717 | 1-*O*-*β*-D-glucopyranosyl sinapate | 387.1802 | 0 | C_17_H_22_O_10_ | Hydroxy cinnamic acid glycosides | 105.06967  121.066  147.06491  387.18321 | [^31^](#_[23]_Santos_MC,) |
|  | 16.97212 | Farnesol | 223.0644 | -0.5 | C_15_H_26_O | Sesquiterpenoids | 121.02569  149.02022  177.05335  223.16804 | [^37^](#_[29]_Greguš_P,) |
|  | 17.8959 | Hinokitiol  (*β*-Thujaplicin) | 165.1257 | 9.3 | C_10_H_12_O_2_ | Tropolones | 91.056  105.06395  119.08852  165.12683 | [^38^](#_[30]_Fujita_K,) |
|  | 18.16072 | 3, 3՛, 4՛, 5-tetrahydroxy-7-methoxyflavone  (Rhamnetin) | 317.1885 | 0.4 | C_16_H_12_O_7_ | Flavonols | 317.1165 | ^11^ |
|  | 19.21533 | Quercetin-3-*O*- glucuronide 6՛՛-*O*-methyl ester | 493.3104 | 0 | C_22_H_20_O_13_ | Flavonoid-3-*O*-glucuronides | 303.05834  493.30888 | ^39^ |
|  | 21.12023 | Daphnetin | 179.144 | -2.1 | C_9_H_6_O_4_ | 7,8-dihydroxy coumarins | 77.03758  123.11621  133.10366  135.11506  179.08454 | ^11^ |
|  | 26.84483 | 3, 5, 7-trihydroxy-4՛-methoxyflavone  (Kaempferide) | 301.1424 | -0.4 | C_16_H_12_O_6_ | Flavonols | 301.1388 | ^11^ |

# **References:**

1. Trendafilova, A., Todorova, M., Nikolova, M., Gavrilova, A. & Vitkova, A. Flavonoid constituents and free radical scavenging activity of *Alchemilla mollis*. *Nat. Prod. Commun.* **6,** 1934578X1100601 (2011).

2. Budzikiewicz, H., Wilson, J. M. & Djerassi, Carl. Mass Spectrometry in structural and stereochemical problems. XXXII. ^1^ pentacyclic triterpenes. *J. Am. Chem. Soc.* **85,** 3688–3699 (1963).

3. Koay, Y. C., Wong, K. C., Osman, H., Eldeen, I. & Asmawi, M. Z. Chemical constituents and biological activities of *Strobilanthes crispus* L. *Nat. Prod*. **7,** 59-64 (2013).

4. El Seadawy, H. M., Abo El Seoud, K. A., Kabbash, A. M., El Aasr, M. & Attia, G. I. Phytochemical and biological investigation of *Urtica Urens* L. growing in Egypt. *The* *International Research Journal of Pharmacy.* **9,** 25–35 (2018).

5. Al-Shammari, L. A., Hassan, W. H. B. & Al-Youssef, H. M. Phytochemical and biological studies of *Carduus pycnocephalus* L. *Journal of Saudi Chemical Society*. **19,** 410–416 (2015).

6. Aljubiri, S. M., Mahmoud, K., Mahgoub, S. A., Almansour, A. I. & Shaker, K. H. Bioactive compounds from *Euphorbia schimperiana* with cytotoxic and antibacterial activities. *South African Journal of Botany.* **141,** 357–366 (2021).

7. Pacifico, S. et al. Antioxidant polyphenolic constituents of *Vitis × labruscana* cv. ‘Isabella’ leaves. *Open Nat. Prod. J.* **5,** 5–11 (2013).

8. Zhang, J. et al. Curcumin targeting NF-κB/Ubiquitin-Proteasome-System axis ameliorates muscle atrophy in triple-negative breast cancer cachexia mice. *Mediators Inflamm.* **2022,** 1–18 (2022).

9. Binsuwaidan, R. et al. In vitro antiviral effect and potential neuroprotection of *Salvadora* *persica* L. stem bark extract against lipopolysaccharides-induced neuroinflammation in mice: LC-ESI-MS/MS analysis of the methanol extract. *Pharmaceuticals.* **16,** 398 (2023).

10. Attallah, N. G. M. et al. Elucidation of the metabolite profile of *Yucca gigantea* and assessment of its cytotoxic, antimicrobial, and anti-inflammatory activities. *Molecules.* **27,** 1329 (2022).

11. Negm, W. A. et al. Promising antifungal activity of *Encephalartos laurentianus* de Wild against *Candida albicans* clinical isolates: in vitro and in vivo effects on renal cortex of adult albino rats. *Journal of Fungi.* **8,** 426 (2022).

12. Elmongy, E. I. et al. Antidiarrheal and antibacterial activities of Monterey cypress phytochemicals: in vivo and in vitro approach. *Molecules.* **27,** 346 (2022).

13. Shang, Z. et al. Profiling and identification of (−)‐epicatechin metabolites in rats using ultra‐high performance liquid chromatography coupled with linear trap‐Orbitrap mass spectrometer. *Drug Test. Anal.* **9,** 1224–1235 (2017).

14. Alqahtani, M. J., Elekhnawy, E., Negm, W. A., Mahgoub, S. & Hussein, I. A. *Encephalartos* *villosus* Lem. displays a strong in vivo and in vitro antifungal potential against *Candida* *glabrata* clinical isolates. *Journal of Fungi.* **8,** 521 (2022).

15. Wilbert, S. M., Ericsson, L. H. & Gordon, M. P. Quantification of jasmonic acid, methyl jasmonate, and salicylic acid in plants by capillary liquid chromatography electrospray tandem mass spectrometry. *Anal. Biochem*. **257,** 186–194 (1998).

16. Negm, W. A. et al. Wound-healing potential of rhoifolin-rich fraction isolated from *Sanguisorba officinalis* roots supported by enhancing re-epithelization, angiogenesis, anti-inflammatory, and antimicrobial effects. *Pharmaceuticals.* **15,** 178 (2022).

17. EL-Shial, E. M., Kabbash, A., El-Aasr, M., El-Feky, O. A. & El-Sherbeni, S. A. Elucidation of natural components of *Gardenia thunbergia* Thunb. leaves: effect of methanol extract and rutin on non-alcoholic fatty liver disease. *Molecules*. **28,** 879 (2023).

18. Attallah, N. G. M. et al. Elucidation of phytochemical content of *Cupressus macrocarpa* leaves: in vitro and in vivo antibacterial effect against methicillin-resistant staphylococcus aureus clinical isolates. *Antibiotics.* **10,** 890 (2021).

19. El Sayed, A. M., Basam, S. M., El-Naggar, E.M. B. A., Marzouk, H. S. & El-Hawary, S. LC–MS/MS and GC–MS profiling as well as the antimicrobial effect of leaves of selected Yucca species introduced to Egypt. *Sci Rep* **10**, 17778 (2020).

20. Du, J. et al. LC-ESI-QTOF-MS/MS profiling and antioxidant activity of phenolics from custard apple fruit and by-products. *Separations*. **8,** 62 (2021).

21. Parejo, I. et al. Separation and characterization of phenolic compounds in fennel ( *Foeniculum vulgare* ) using liquid chromatography−negative electrospray ionization tandem mass spectrometry. *J. Agric. Food Chem.* **52,** 3679–3687 (2004).

22. Liguori, A., Mascaro, P., Porcelli, B., Sindona, G. & Uccella, N. Identification of caffeine and its metabolites in human urine extracts by electron impact ionization tandem mass spectrometry. *Organic Mass Spectrometry.* **26,** 608–612 (1991).

23. Hegazy, M. M. et al. Antitrypanosomal, antitopoisomerase-i, and cytotoxic biological evaluation of some african plants belonging to crassulaceae; chemical profiling of extract using UHPLC/QTOF-MS/MS. *Molecules.* **27,** 8809 (2022).

24. Scorrano, S. et al. Anthocyanins profile by Q-TOF LC/MS in *Myrtus communis* berries from Salento Area. *Food Anal. Methods.* **10,** 2404–2411 (2017).

25. Mahrous, F., Mohammed, H., Sabour, R. & Ismail, L. LC-ESI-QTOF-MS/MS of *Holoptelea* *integrifolia* (Roxb.) Planch. leaves and In silico study of phenolic compounds’ antiviral activity against the HSV1 virus. *Azhar International Journal of Pharmaceutical and Medical Sciences.* **1,** 91–101 (2021).

26. Alotaibi, B. et al. Antimicrobial activity of *Brassica rapa* L. Flowers extract on gastrointestinal tract infections and antiulcer potential against indomethacin-induced gastric ulcer in rats supported by metabolomics profiling. *J. Inflamm. Res.* **14,** 7411–7430 (2021).

27. Kadam, D., Palamthodi, S. & Lele, S. S. LC–ESI-Q-TOF–MS/MS profiling and antioxidant activity of phenolics from *L. Sativum* seedcake. *J. Food Sci. Technol.* **55,** 1154–1163 (2018).

28. Mohammed, H. A. et al. Phytochemical profiling, in vitro and in silico anti-microbial and anti-cancer activity evaluations and Staph GyraseB and *h*-TOP-IIβ receptor-docking studies of major constituents of *Zygophyllum coccineum* L. aqueous-ethanolic extract and its subsequent fractions: an approach to validate traditional phytomedicinal knowledge. *Molecules.* **26,** 577 (2021).

29. Binsuwaidan, R. et al. Antibacterial activity and wound healing potential of *Cycas thouarsii* R.Br *n*-butanol fraction in diabetic rats supported with phytochemical profiling. *Biomedicine & Pharmacotherapy.* **155,** 113763 (2022).

30. Bochi, V. C., Godoy, H. T. & Giusti, M. M. Anthocyanin and other phenolic compounds in Ceylon gooseberry (*Dovyalis hebecarpa*) fruits. *Food Chem.* **176,** 234–243 (2015).

31. Santos, M. C. et al. UPLC-MS for identification of quercetin derivatives in *Cuphea* *glutinosa* Cham. &amp; Schltdl (Lythraceae) and evaluation of antifungal potential. *Curr. Pharm. Anal.* **14,** 586–594 (2018).

32. Chen, S. et al. Simultaneous qualitative assessment and quantitative analysis of flavonoids in various tissues of lotus (*Nelumbo nucifera*) using high performance liquid chromatography coupled with triple quad mass spectrometry. *Anal. Chim. Acta.* **724,** 127–135 (2012).

33. Gouda, H., Morsy, A., Youssef, A., Tolba, I. & Hassan, G. Phytochemical Profile and Antimicrobial Assessment of *Abutilon fruticosum* Guill. &amp; Perr. Growing in Gebel Elba, Egypt. *Egypt. J. Chem*. **65,** 1299–1305 (2022).

34. Upreti, M. et al. Photostability study of natural high-potency sweetener monatin in a model beverage system and characterisation of the degradation products. *Food Chem.* **131,** 413–421 (2012).

35. Shen, J. et al. Development of a HPLC-MS/MS method to determine 11 bioactive compounds in Tongmai Yangxin Pill and application to a pharmacokinetic study in rats. *Evidence‐Based Complementary and Alternative Medicine.* **2018,** 1–11 (2018).

36. Smyth, T. J., Ramachandran, V. N., McGuigan, A., Hopps, J. & Smyth, W. F. Characterisation of nicotine and related compounds using electrospray ionisation with ion trap mass spectrometry and with quadrupole time‐of‐flight mass spectrometry and their detection by liquid chromatography/electrospray ionisation mass spectrometry. *Rapid Communications in Mass Spectrometry.* **21,** 557–566 (2007).

37. Greguš, P. et al. Ultra high performance liquid chromatography tandem mass spectrometry analysis of quorum-sensing molecules of *Candida albicans*. *J. Pharm Biomed. Anal.* **53,** 674–681 (2010).

38. Fujita, K. et al. A novel synthetic pathway for tropolone ring formation via the olefin monoterpene intermediate terpinolene in cultured *Cupressus lusitanica* cells. *J. Plant Physiol*. **171,** 610–614 (2014).

39. Hassan, A. R. Chemical profile and cytotoxic activity of a polyphenolic-rich fraction from *Euphorbia dendroides* aerial parts. *South African Journal of Botany.* **147,** 332–339 (2022).
